# Population Genetic Structure of a Sandstone Specialist and a Generalist Heath Species at Two Levels of Sandstone Patchiness across the Strait of Gibraltar

**DOI:** 10.1371/journal.pone.0098602

**Published:** 2014-05-30

**Authors:** Manuel Jesús Gil-López, José Gabriel Segarra-Moragues, Fernando Ojeda

**Affiliations:** 1 Departamento de Biología, Universidad de Cádiz, Puerto Real, Cádiz, Spain; 2 Centro de Investigaciones sobre Desertificación, Consejo Superior de Investigaciones Científicas, Moncada, Valencia, Spain; University of Innsbruck, Austria

## Abstract

Many habitat specialist species are originally composed of small, discontinuous populations because their habitats are naturally fragmented or patchy. They may have suffered the long-term effects of natural patchiness. Mediterranean heathlands, a representative habitat in the Strait of Gibraltar region, are associated with nutrient-poor, acidic sandstone soils. Sandstone soil patches in the African side of the Strait (Tangier) are, in general, smaller and more scattered than in the European side (Algeciras). In this study, we analyze the effect of this sandstone patchiness on the population genetic diversity and structure of two *Erica* species from these Mediterranean heathlands that differ in their edaphic specificity, *E. australis*, sandstone specialist, and *E. arborea*, generalist. Average levels of within-population genetic diversity and gene flow between populations were significantly lower in Tangier (high sandstone patchiness) than in Algeciras (low patchiness) for the sandstone specialist, whereas no differences between both sides of the Strait were detected in the edaphic generalist. Since most endemic species in Mediterranean heathlands of the Strait of Gibraltar are sandstone specialists, these results highlight an increased vulnerability to loss of genetic diversity and local extinction of the heathland endemic flora in the Tangier side of the Strait of Gibraltar.

## Introduction

Plant populations in small habitat fragments have frequently smaller population sizes and experience a higher degree of isolation among populations than those from continuous or less fragmented habitats [1]. The smaller and more isolated the populations, the more liable they are to demographic stochasticity, to genetic depletion and, ultimately, to local extinction [2]–[4]. These small, fragmented populations are also highly vulnerable to edge effects, habitat degradation or to catastrophic disturbance events, either natural or human-induced [5], [6].

Genetic diversity in a plant population is lost mostly by the effect of genetic drift and by reduced genetic communication (i.e. low gene flow and migration) among populations [7]. The loss of genetic diversity negatively affects the adaptability of populations to environmental changes as it constitutes the basic evolutionary substrate and therefore lessens their chances of survival [8], [9]. Gene flow reduces the risk of extinction of small populations either by avoiding genetic erosion through the input of new alleles mainly via seed dispersal or by recolonization after a local extinction event [10]–[11]. Therefore, gene flow allows the long-term persistence of small populations and it is a critical process for the management and conservation of threatened species in fragmented or degraded habitats [3], [12].

Habitat degradation and fragmentation have indeed become major research topics in both population genetics and conservation biology [13], and many scientific contributions report the effects of anthropogenic fragmentation on the population genetic structure of species that previously occupied large, continuous habitats (e.g. [3], [14], [15]). However, fragmentation has not always a human-induced cause: many habitat specialist species are originally composed of small, discontinuous populations because their habitats are naturally fragmented or patchy [16]. Those habitat specialist species may have undergone the long-term effects of natural patchiness and their populations might exhibit low genetic diversity within populations and strong genetic structure among populations (e.g. [17]; but see [18]). As a result, a specialist species confined to a naturally patchy habitat would be inherently more sensitive to further fragmentation and/or degradation of the habitat than a generalist species occurring within and outside those habitat patches [19], [20]. It is thus crucial to ascertain whether natural habitat patchiness does actually affect the genetic diversity of populations of habitat specialist species.

**Figure 1:**
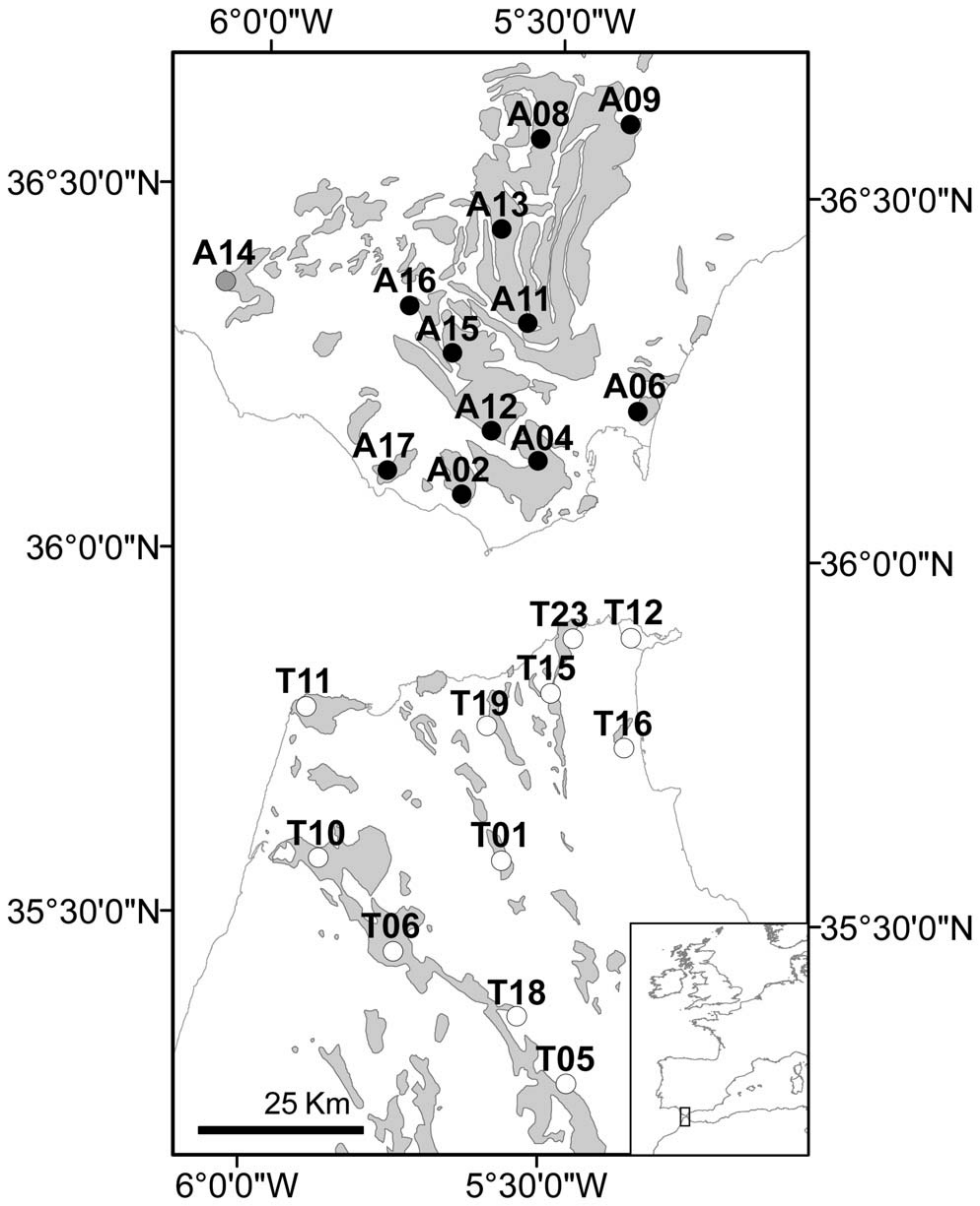
Geographical location of sampling sites of *E. australis* and *E. arborea*. At each site in Algeciras (black dots) and Tangier (white dots), one population of each species was sampled (except in A14, grey dot, where only *E. australis* was sampled, see Table 1). Grey shaded patches indicate the presence of nutrient-poor, highly acidic, sandstone soils.

Many endemic, and/or endangered plant species in the Mediterranean Basin, one of the world’s biodiversity hotspots, are habitat specialists − edaphic and/or orographic − restricted to uncommon, patchy habitats. Mediterranean heathlands of the Strait of Gibraltar region, at the western end of the Mediterranean, harbour an endemic-rich flora composed mainly of edaphic specialist species, restricted to nutrient-poor, highly acidic, sandstone soils (sandstone specialists; [21], [22]). Mediterranean heathlands on either side of the Strait of Gibraltar are very similar from both floristic and landscape points of view [23]. However, the absolute number and relative abundance of sandstone endemics in heathland communities of the African side of the Strait (hereafter *Tangier*) are lower than those from the European side (hereafter *Algeciras*) [22]. This seems to be a direct outcome of a striking feature of the Strait of Gibraltar: the generally smaller size and more isolated pattern of the sandstone patches in Tangier (Fig. 1; [22]). The overall higher sandstone patchiness in Tangier might limit gene flow and recolonization among populations of sandstone specialists − most of them endemics − thus making them highly prone to local extinction [19] in comparison with the generally more continuous and larger sandstone patch sizes in Algeciras (Fig. 1).

Here we explore whether an overall high level of sandstone patchiness is associated to low levels of within-population genetic diversity and of gene flow between populations of sandstone specialist species. To do so, we studied the genetic diversity and population structure of two heath species, *Erica australis* L. and *E. arborea* L. by means of microsatellite markers. These two species are abundant in Mediterranean heathlands from both Algeciras and Tangier, but they differ notably in their edaphic (sandstone) specificity: while *E. australis* is a sandstone specialist, virtually restricted to sandstone-derived soils on both sides of the Strait, *E. arborea* has a broader edaphic spectrum [24]. We compared population genetic diversity indices, including relative levels of gene flow among populations, for each species between the two sides of the Strait as surrogates for overall low (Algeciras) and high (Tangier) levels of sandstone patchiness. Since an overall high patchiness would affect sandstone specialists more strongly than generalists, we expect to find lower levels of genetic diversity and gene flow in Tangier than in Algeciras populations for *E. australis*, but not for *E. arborea*.

This study provides valuable insights into the differential effects of natural habitat (sandstone) patchiness on the genetic diversity and structure of populations of habitat specialist and generalist species. Considering that most endemic species from these Mediterranean heathlands are sandstone specialists [21], [22], [25], this study also allows us to highlight a presumably higher vulnerability to loss of genetic diversity and ultimately local extinction of the heathland endemic flora in the African side of the Strait of Gibraltar (i.e. Tangier), probably as a consequence of its overall higher sandstone patchiness. From a conservation perspective, this knowledge could help focus appropriate conservation on those patchy communities to better maintain the high biodiversity and uniqueness of Mediterranean heathlands across the Strait of Gibraltar region.

## Materials and Methods

### Ethic Statement

Necessary permits for fieldwork and sampling were obtained through the University Abdelmalek-Essaadi (Morocco) and the *Junta de Andalucía* (Spain). None of the two species used in this study is endangered or red-listed.

### Study Species

*Erica australis* and *E. arborea* (Ericaceae) are two relatively abundant heath species in Mediterranean heathlands on acid, nutrient-poor, Oligo-Miocene sandstone soils at both sides of the Strait of Gibraltar [25]. They are both diploid (2*n* = 2x = 24; [26]) and monophyletic [27], [28], and may be considered genetically related as inferred from a high transferability rate from SRR markers from a South African congener [29]. They are also morphologically and ecologically similar (e.g. long-lived, woody plants with ericoid leaves, acidophilous and post-fire resprouters; [24], [30], [31]. However, they differ notably in their edaphic specificity: *E. australis* is a sandstone specialist, restricted to highly acidic, sandstone soils, whereas *E. arborea* has a broader edaphic spectrum. Within the regional context of this study (i.e. Strait of Gibraltar region), *E. australis* is virtually restricted to highly acidic, aluminium-rich sandstone soil patches whereas *E. arborea* occurs both within sandstone patches and out in the surrounding matrix on different, non-acid soils [24]. They also differ in the amplitude of their geographical range: *E. australis* is endemic to the western end of the Mediterranean basin (western third of the Iberian Peninsula and NW tip of Africa), whereas *E. arborea* has a much wider, circum-Mediterranean and eastern African distribution [27], [30].

Regarding aspects of reproductive biology, both species have small, hermaphroditic flowers, pink-coloured in *E. australis* and whitish in *E. arborea*. They are insect pollinated, with pollen shed in tetrads [32]. *Erica australis* sets flowers in late-winter to early-spring (December to April), whereas *E. arborea* is a spring-blooming species (March to May; [33]). Both species produce small, oval seeds that lack any kind of appendages for long distance dispersal. *Erica australis* seeds are larger (0.9–1.1 mm length) than those of *E. arborea* (0.4–0.5 mm; [34]). Such small seeds may be wind-dispersed but only over limited distances (less than 100 m; [35]). *Erica australis* presents a small caruncle [32], which might indicate also short-distance, ant-dispersal.

### Population Sampling, DNA Extraction and Microsatellite Amplification

Twenty-two localities (11 from Algeciras and 11 from Tangier) where *E. australis* and *E. arborea* co-occurred, plus one locality in Algeciras where only *E. australis* was found, were sampled (Table 1, Fig. 1). Population sampling consisted of fresh leaves from up to 30 individuals per population (spaced at least 10 m from each other), except in four populations of *E. australis* and three populations of *E. arborea* where this number could not be reached (Table 1). Thus, the total sampling included 676 individuals of *E. australis* (357 from Algeciras and 319 from Tangier) and 631 individuals of *E. arborea* (308 from Algeciras and 323 from Tangier). Samples were dried in silica gel and stored at room temperature until DNA extraction. Dry leaf material, approximately 100 mg per sample, was reduced to fine powder using stainless steel beads on a Mixer Mill MM400 cell disrupter (Retsch, Llanera, Spain). DNA was extracted using SpeedTools plant DNA extraction kit (Biotools, Madrid, Spain), and eluted in 50 µl in Tris-EDTA 0.1×buffer.

**Table 1:** Geographical position of the sampling sites, population size estimation and genetic diversity indices of the twenty-three *E. australis* and twenty-two *E. arborea* populations for seven and eight microsatellite loci, respectively.

Populations	Latitude	Longitude	Altitude (m)	*Erica australis*					*Erica arborea*				
				*N* 1	*A* 1	*H*_O_ 1	*H*_E_ 1	*F*_IS_ 1	*N* 1	*A* 1	*H*_O_ 1	*H*_E_ 1	*F*_IS_ 1
**Algeciras**													
**A02-Sierra de Tarifa**	36°04′59″N	05°39′17″W	450	30	11.14	0.690	0.786	+0.1235^***^	30	6.62	0.641	0.606	−0.0598^ns^
**A04-Sierra de la Palma**	36°07′55″N	05°31′40″W	450	30	10.57	0.590	0.776	+0.2424^***^	30	7.25	0.566	0.611	+0.0740^***^
**A06-Sierra Carbonera**	36°12′10″N	05°21′37″W	270	29	8.14	0.616	0.743	+0.1747^***^	15	5.75	0.525	0.579	+0.0968^*^
**A08-Puerto Galis**	36°34′26″N	05°32′12″W	600	30	9.57	0.652	0.737	+0.1173^***^	30	6.00	0.558	0.623	+0.1056^*^
**A09-Cortes**	36°35′50″N	05°23′07″W	705	30	8.57	0.614	0.712	+0.1399^***^	30	6.37	0.520	0.622	+0.1659^***^
**A11-Murta**	36°19′14″N	05°33′02″W	360	30	10.00	0.657	0.751	+0.1276^***^	30	5.62	0.529	0.572	+0.0759^***^
**A12-Sierra del Niño**	36°10′17″N	05°36′25″W	665	30	9.71	0.609	0.704	+0.1367^***^	23	6.12	0.559	0.623	+0.1038^ns^
**A13-La Peguera**	36°26′56″N	05°35′57″W	385	30	10.71	0.609	0.745	+0.1849^***^	30	6.75	0.604	0.605	+0.0018^ns^
**A14-Pago del Humo**	36°22″00″N	06°03′53″W	70	28	8.85	0.627	0.778	+0.1964^***^	–	–	–	–	–-
**A15-El Cuervo**	36°16′37″N	05°40′37″W	436	30	11.14	0.695	0.778	+0.1085^*^	30	6.25	0.591	0.605	+0.0224^**^
**A16-Sierra de la Momia**	36°20′26″N	05°45′06″W	176	30	9.71	0.652	0.761	+0.1449^***^	30	6.12	0.558	0.577	+0.0343^ns^
**A17-Sierra de la Plata**	36°06′49″N	05°46′52″W	359	30	10.85	0.638	0.755	+0.1580^**^	30	6.75	0.650	0.613	−0.0610^ns^
**Tangier**													
**T01-El Fendek**	35°33′53″N	05°33′44″W	554	30	10.71	0.685	0.754	+0.0929^***^	30	5.62	0.550	0.589	+0.0673^***^
**T05-Jbel Bouhachem**	35°16′37″N	05°27′14″W	1000	30	9.14	0.605	0.714	+0.1560^***^	30	6.12	0.571	0.597	+0.0458^ns^
**T06-Jbel Habib**	35°27′11″N	05°44′56″W	835	30	9.28	0.704	0.756	+0.0691^***^	30	5.37	0.525	0.534	+0.0179^ns^
**T10-Cuesta Colorada**	35°34′44″N	05°52′45″W	250	30	8.42	0.590	0.688	+0.1438^***^	30	5.75	0.541	0.573	+0.0563^*^
**T11-Cap Spartel**	35°47′10″N	05°54′24″W	295	29	10.57	0.625	0.707	+0.1176^***^	30	6.25	0.625	0.592	−0.0558^ns^
**T12-Ceuta**	35°54′25″N	05°21′25″W	145	20	6.42	0.614	0.703	+0.1295^**^	30	6.75	0.525	0.613	+0.1466^***^
**T15-Taghramet**	35°48′47″N	05°29′47″W	360	30	10.14	0.628	0.672	+0.0659^***^	30	6.00	0.554	0.581	+0.0474^*^
**T16-Jbel Zem-Zem**	35°44′25″N	05°22′12″W	230	30	8.14	0.505	0.554	+0.0915^ns^	30	7.37	0.537	0.614	+0.1262^ns^
**T18-Beni-Yder**	35°22′07″N	05°32′22″W	520	30	10.00	0.581	0.707	+0.1807^***^	30	5.37	0.562	0.584	+0.0371^ns^
**T19-Mellousa**	35°45′59″N	05°36′06″W	440	30	9.00	0.619	0.704	+0.1227^***^	23	6.00	0.538	0.574	+0.0642^ns^
**T23-Punta Cires**	35°53′20″N	05°27′39″W	260	30	10.14	0.633	0.707	+0.1063^***^	30	6.87	0.575	0.611	+0.0598^ns^

1 *N* = sample size; *A* = mean number of alleles per locus; *H*_O,_ *H*_E_ = observed and unbiased expected heterozygosity, respectively; *F*_IS_ = inbreeding coefficient. ns, not significant; *p<0.05; **p<0.01; ***p<0.001.

Amplification of microsatellite loci followed Segarra-Moragues *et al*. [29], [36]. Seven microsatellite loci (Ecoc108, Ecoc117, Ecoc132, Ecoc137, Ecoc142, Ecoc431 and Ecoc446) were amplified in *E. australis*, and eight (the same as for *E. australis* plus Ecoc115) in *E. arborea*. These microsatellite loci were unlinked [29] and polymorphic in both species. Fifteen percent of the 1307 genotyped individuals were included as duplicates to check for possible genotyping errors. All of them showed identical allelic profiles to their corresponding original samples.

PCR products were electrophoresed in an ABI3730 automated sequencer (Applied Biosystems, Madrid, Spain) using LIZ500 as internal lane size standard. Assignment of fragments to allele classes was carried out with Genemarker version 1.97 software (Softgenetics, State College, PA). Genotypic matrices were deposited at DRYAD (http://datadryad.org/) under accession number doi:10.5061/dryad.bj70k.

### Population Genetic Analyses

GENETIX v. 4.05 [37] was used to estimate allele frequencies, mean number of alleles per locus (*A*), and observed (*H*_O_) and unbiased expected (*H*_E_) heterozygosities [38]. Wright’s *F*-statistics were estimated according to Weir & Cockerham [39] using GENEPOP′007 [40] and tested for significance using Fisher’s exact tests. This latter software was also used to estimate the Maximum likelihood frequency of null alleles and 95% confidence intervals for null allele frequencies according to Dempster *et al*. [41].

To evaluate, for each species, whether population diversity indices differed between Algeciras and Tangier groups of populations, average allelic richness per locus (*A* ^*^) estimated according to the rarefaction method of Hurlbert [42] adapted by El Mousadik & Petit [43], average observed heterozygosity (*H*_O_), average genetic diversity within populations (*H*_S_), and inbreeding coefficient (*F*_IS_), were compared using FSTAT version 2.9.3.2 [44] and tested for significance using 10,000 permutations. This same software was used to check for significant differences in population differentiation (i.e. average *F*_ST_ values) for each species between Algeciras and Tangier.

Bayesian analyses in STRUCTURE v. 2.1 [45], [46] were used to estimate the population genetic structure and to infer the most likely number of genetic clusters (*K*) in both heath species. STRUCTURE assigns individuals to the *K* different genetic clusters based on allele frequencies at each locus. Estimated number of *K* clusters ranged from 1 to 21, and analyses were based on an admixture ancestral model with correlated allele frequencies. In each run of the program Monte Carlo Markov Chain (MCMC) and burn-in period length consisted of 1.2×10^6^ and 8×10^5^ iterations, respectively. The amount of variation of the likelihood was evaluated by carrying out ten runs for each *K*. The most likely number of genetic clusters (*K*) was estimated following Evanno *et al*. [47], which uses an *ad hoc* parameter (Δ*K*) to estimate the rate of change of likelihood values between successive *K* values. POPULATIONS version 1.2.3. beta [48] was used to compute pairwise *D*_A_ genetic distances [49] between populations of each species. These *D*_A_ genetic distance matrices were used to compute eigenvalues and eigenvectors to perform Principal Coordinates Analyses (PCoA) for each species. Minimum Spanning Trees (MST) using *D*_A_ distance matrices were constructed with NTSYSpc version 2.1 [50] and were superimposed onto the 2D-PCoA plots. MST computes minimum-length pairwise connections between points. When superimposed onto PCoA plots it helps detecting local distortions (i.e. pairs of points which look close together in the PCoA but actually are far apart if other dimensions). Isolation by distance (IBD) was estimated separately for *E. arborea* and *E. australis* at each side of the Strait by matrix correlation analyses using a matrix of log-transformed pairwise geographical distances between populations and a matrix of a pairwise linearized *F*_ST_ values (i.e., *F*_ST_/(1 - *F*_ST_); [51]) computed with ARLEQUIN v. 3.11 [52]. Significance of the correlation was tested for each species and side of the Strait with Mantel test (1000 permutations) using NTSYSpc. Then, ANCOVAs were used to test for parallelism of the resulting IBD slopes in Algeciras and Tangier within each species. Finally, gene flow between population pairs was also estimated separately for each species at each side of the Strait as the effective number of migrants per generation (*N*_m_), obtained directly from *F*_ST_ values by using Wright’s [53] island model. Although these values should not be taken as accurate estimates of numbers of migrants, the comparison of average *N*_m_ values between populations at each of the two sides of the Strait may still be useful to explore for each species the existence of a decrease in gene flow associated with higher patchiness. For the sake of this study, we focused on geographical distances separating pairs of populations smaller than 30 km in order to factor out or minimize possible geographical effects other than sandstone patchiness.

## Results

### Genetic Variation in *E. australis* and *E. arborea* Populations

The seven microsatellite loci amplified a total of 167 alleles in *Erica australis* (Table S1). Number of alleles per locus ranged from 10 (Ecoc446) to 41 (Ecoc 117), with a mean of 23.85±10.02 alleles per locus. Mean number of alleles per locus ranged from a minimum of 6.42 from the Tangier population T12 to a maximum of 11.14 in the Algeciras populations A02 and A15 (Table S1). Average observed heterozygosities (*H*_O_) ranged from 0.505 (population T16) to 0.704 (population T06), and unbiased expected heterozygosities (*H*_E_) ranged from 0.554 (population T16) to 0.786 (population A02; Table 1). In *E. arborea* a total of 108 alleles were scored from eight microsatellite loci (Table S2). Number of alleles per locus ranged from 2 (Ecoc117) to 30 (Ecoc446) with an average of 13.50±9.16 alleles. Mean number of alleles per population in *E. arborea* ranged from a minimum of 5.37 in the Tangier populations T06 and T18 to a maximum of 7.37 in the Tangier population T16. Average observed heterozygosities ranged from 0.520 (population A09) to 0.650 (population A17), and unbiased expected heterozygosities ranged from 0.534(population T06) to 0.623 (populations A08 and A12; Table 1).

Significant deviation towards heterozygote deficiency (i.e. positive *F*_IS_) was found in 22 out of the 23 populations studied of the sandstone specialist *E. australis* (Table 1). A similar pattern was also detected in the edaphic generalist *E. arborea*, although positive *F*IS values in this species were significant only in 10 out of the 22 populations (Table 1). High *F*_IS_ values may be influenced by biological and methodological factors including inbreeding, local population substructure (i.e wahlund effect) and null alleles. Estimation of null allele frequencies gave significant results (i.e. lower 95%CI allele frequency >0.05) in 24 out of 161 and 39 out of 176 locus × population combinations in *E. australis* and *E. arborea*, respectively. Most of these significant combinations were concentrated in one locus (Ecoc431, 15 populations) in *E. australis* and two loci (Ecoc132, 22 populations and Ecoc431, 15 populations) in *E. arborea*. Although the actual presence of null alleles in the dataset may only be confirmed by progeny analyses, our large sampling sizes provided indirect evidence that the frequencies of null alleles could have been overestimated by Dempster et al.’s (1977) method used in GENEPOP. As an example, in locus Ecoc431 we should have found ca. 20 null homozygotes out of 446 individuals genotyped in 15 populations that showed significant estimated frequencies of null alleles, but none was found. Therefore, null alleles, if at all present, should have little impact in our datatset. Besides, repeated estimation of genetic diversity indices and population relationships did not change significantly after removing the two loci (data not shown). Accordingly, high *F*_IS_ in these heath species may be related to pollen dispersal system favouring inbreeding and/or to population substructure, as it has been reported in other *Erica* species [36], [54].

Although average within-population genetic diversity indices (A*, *H*_O_ and *H*_S_) in both species were slightly lower in Tangier, where sandstone patchiness is more prominent (Fig. 1), only average *H*_S_ was significantly lower in Tangier for the sandstone specialist *E. australis*, (Table 2). In this species, Algeciras populations showed a significantly higher inbreeding coefficient (*F*_IS_) than Tangier ones, whereas no significant differences were detected in the edaphic generalist *E. arborea* (Table 2).

**Table 2:** Comparison of mean genetic diversity values between Algeciras and Tangier populations of *Erica australis* and *Erica arborea.*

	Erica australis			Erica arborea		
	Algeciras (N = 12)	Tangier (N = 11)	P-value	Algeciras (N = 11)	Tangier (N = 11)	P-value
A* 1	8.683	8.119	0.103	5.419	5.212	0.186
**H_O_** 2	0.638	0.618	0.291	0.576	0.555	0.213
**H_S_** 2	0.754	0.698	**0.001**	0.605	0.589	0.082
**F_IS_**	0.155	0.116	**0.024**	0.048	0.056	0.758
**F_ST_**	0.057	0.070	0.278	0.026	0.038	0.186

1 A* = allelic richness calculated after the rarefaction method of El Mousadik and Petit (1996).

2 *H*_O_, *H*_S_, average values of observed heterozygosity and genetic diversity within populations, respectively. *N* = Number of sampled populations. Significant values based on 10,000 permutations are indicated in bold.

### Population Genetic Structure of *E. arborea* and *E. australis*

Pairwise *F*_ST_ values in *E. australis* ranged from 0.010 (pair A02–A15) to 0.164 (T05–T16), whereas for *E. arborea* they ranged from zero (pair T01–T05) to 0.106 (pair T06–T16). Average *F*_ST_ values were significantly different from zero in the two species, but higher in the sandstone specialist *E. australis* (average *F*_ST_ = 0.074) than in the edaphic generalist *E. arborea* (average *F*_ST_ = 0.039). The comparison of average *F*_ST_ values between the two sides of the Strait revealed for the two species a slightly stronger genetic differentiation in Tangier populations, although these differences were not significant in either species (Table 2).

**Figure 2:**
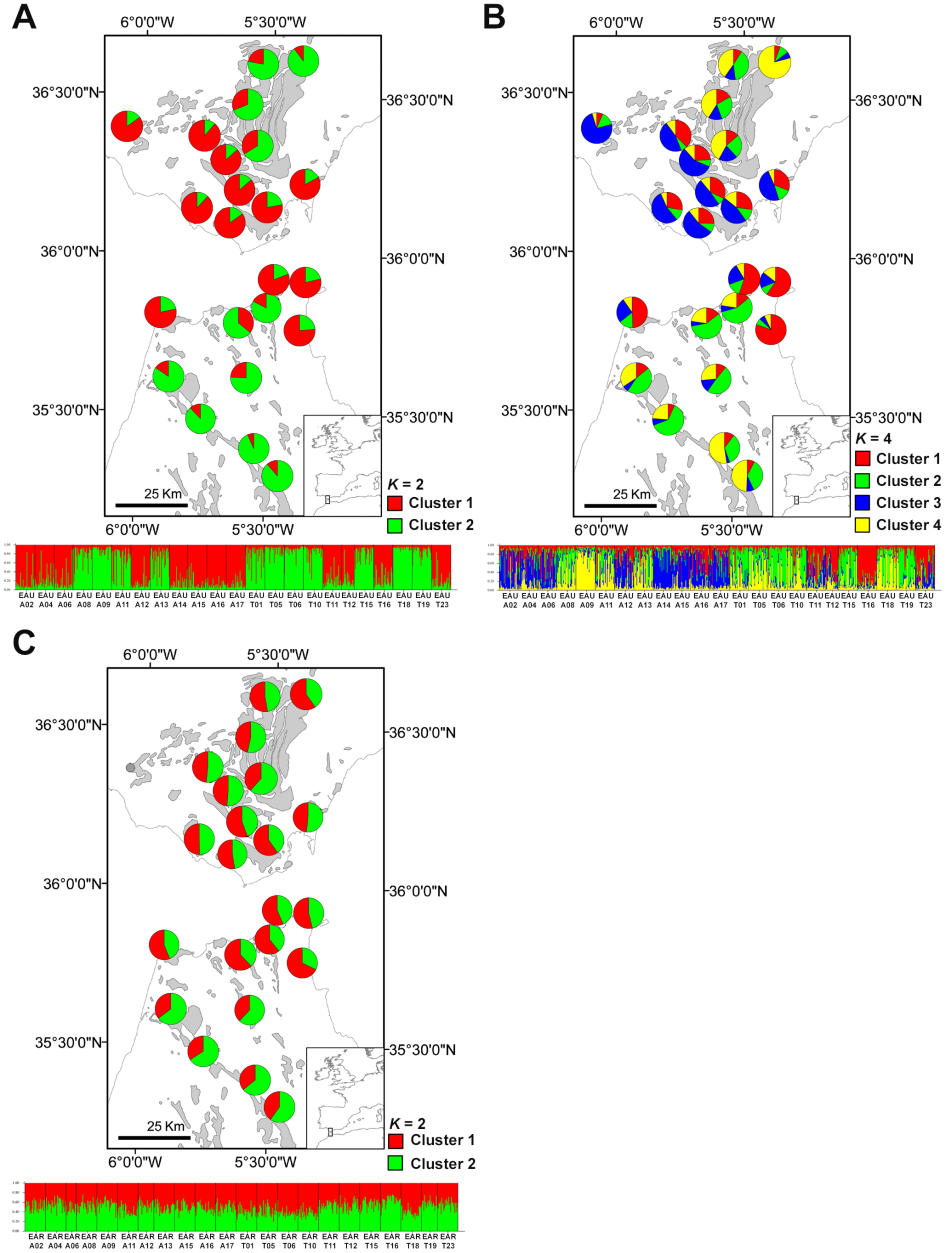
Bayesian analyses of genetic structure from twenty-three populations of *E. australis* (a, b) and twenty-two of *E. arborea* (c), respectively. Mean proportion membership of each population to the predefined, *K* = 2 and *K* = 4 (only *E. australis*) clusters with the highest Δ*K* values obtained following Evanno *et al*. (2005). Proportion of membership of individuals for predefined clusters in both species.

The method of Evanno *et al*. [47] for the estimation of the most likely number of genetic clusters (*K*) revealed a maximum modal value of Δ*K* for *K = *2 for both *E. australis* and *E. arborea* (see Figure S1). These values were Δ*K* = 1794.02 and Δ*K = *24.43 for *E. australis* and *E. arborea*, respectively (Fig. S1). A second maximum modal value of Δ*K = *263.17 was found for *K = *4 for *E. australis* (Fig. S1). The proportion of membership of the populations to each of the two genetic clusters (*K = *2) in *E. australis* did not correspond to the geographical membership of the populations, with some populations of each side showing a higher proportion of membership to cluster 1 and others to cluster 2 (Fig. 2a). For *K = *4, populations of *E. australis* from Algeciras showed a higher proportion of membership to clusters 3 and 4, whereas populations from Tangier showed a higher proportion of membership to clusters 1, 2 and 4 (Fig. 2b). Interestingly, populations of the sandstone specialist *E. australis* from the high sandstone patchiness area (Tangier) showed higher proportion of membership to more different genetic clusters than populations from the low patchiness one (Algeciras). On the other hand, in the generalist *E. arborea*, populations and individuals showed an almost symmetrical membership to both genetic clusters revealed at *K* = 2 in either Tangier or Algeciras (Fig. 2c). The method of Evanno *et al*. [47] is computationally constrained to detect *K*>2 genetic clusters; the most likely outcome for *E. arborea* is in fact *K* = 1 [55] which cannot be tested with this method. However, this homogeneous admixture pattern altogether with the low Δ*K* value obtained in *E. arborea* is indicative of an absence of population genetic structure (i.e. *K* = 1) throughout the Strait of Gibraltar and within Tangier and Algeciras [45] suggesting a weaker effect of patchiness in this species (Fig. 2c).

**Figure 3:**
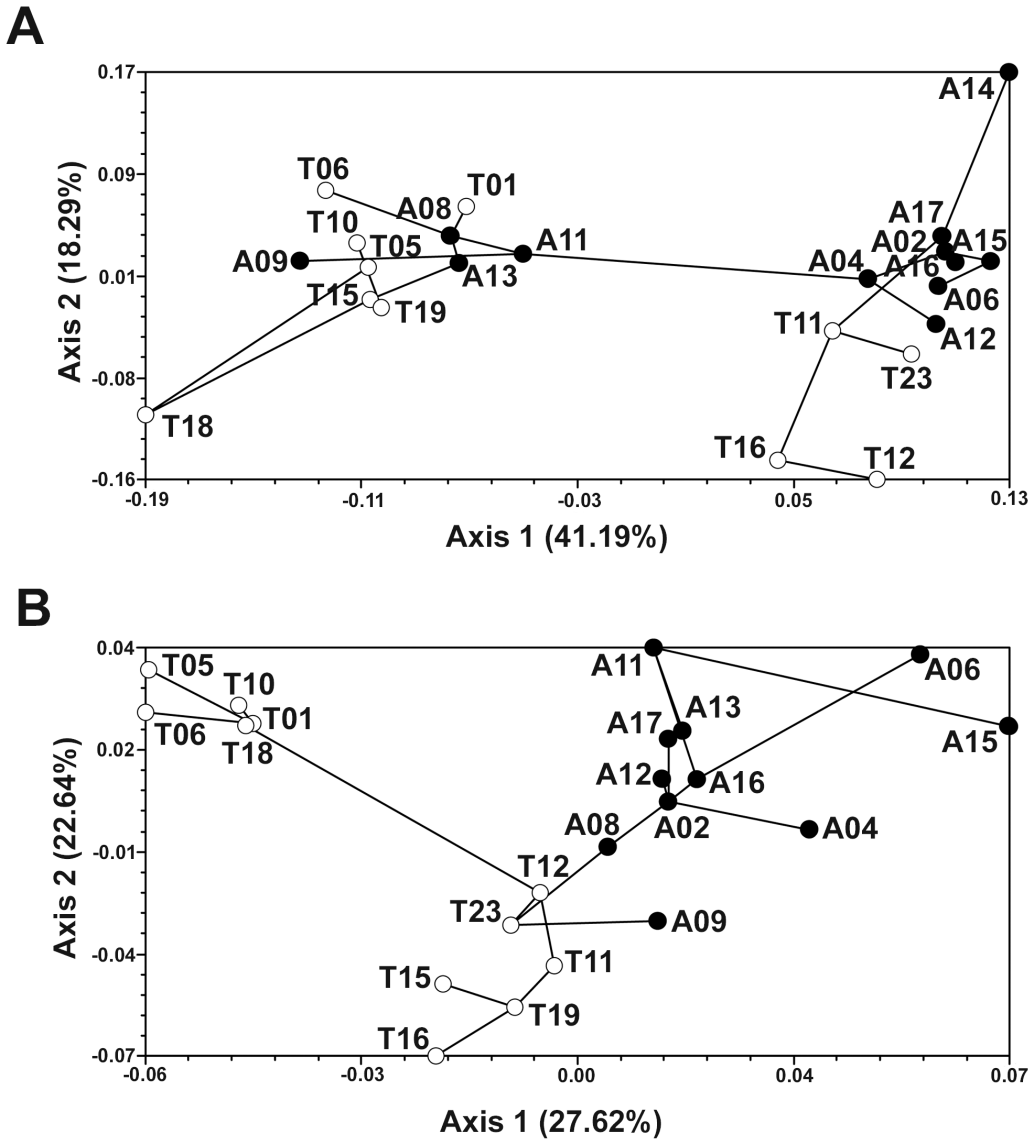
Principal Coordinates Analyses (PCoA). It has been constructed with *D*_A_ genetic distance of Nei *et al*. (1983), showing the association among populations of *E. australis* (a) and *E. arborea* (b). Black dots and white dots represent Algeciras and Tangier sites, respectively. Minimum Spanning Trees (MST) superimposed onto PCoA plots show the minimum length links between populations.

The two-dimensional Principal Coordinates Analyses (PCoA)+MST revealed similar results to those from STRUCTURE, and also showed direct links between populations (Figs. 3a–b). In *E. australis* the PCoA separated two population groups along the first axis, both of them including Algeciras and Tangier populations (Fig. 3a). In *E. arborea*, by contrast, the spatial arrangement of the populations in the PCoA was somewhat consistent with their geographical membership (Fig. 3b). The largest group was composed of two subclusters of populations. One of these included all Algeciras populations, with the exception of population A09, which appeared derived from the second subcluster that included all northernmost coastal Tangier populations. This second subcluster was then linked to another, smaller group composed of the five southernmost Tangier populations (Fig. 3b).

**Figure 4:**
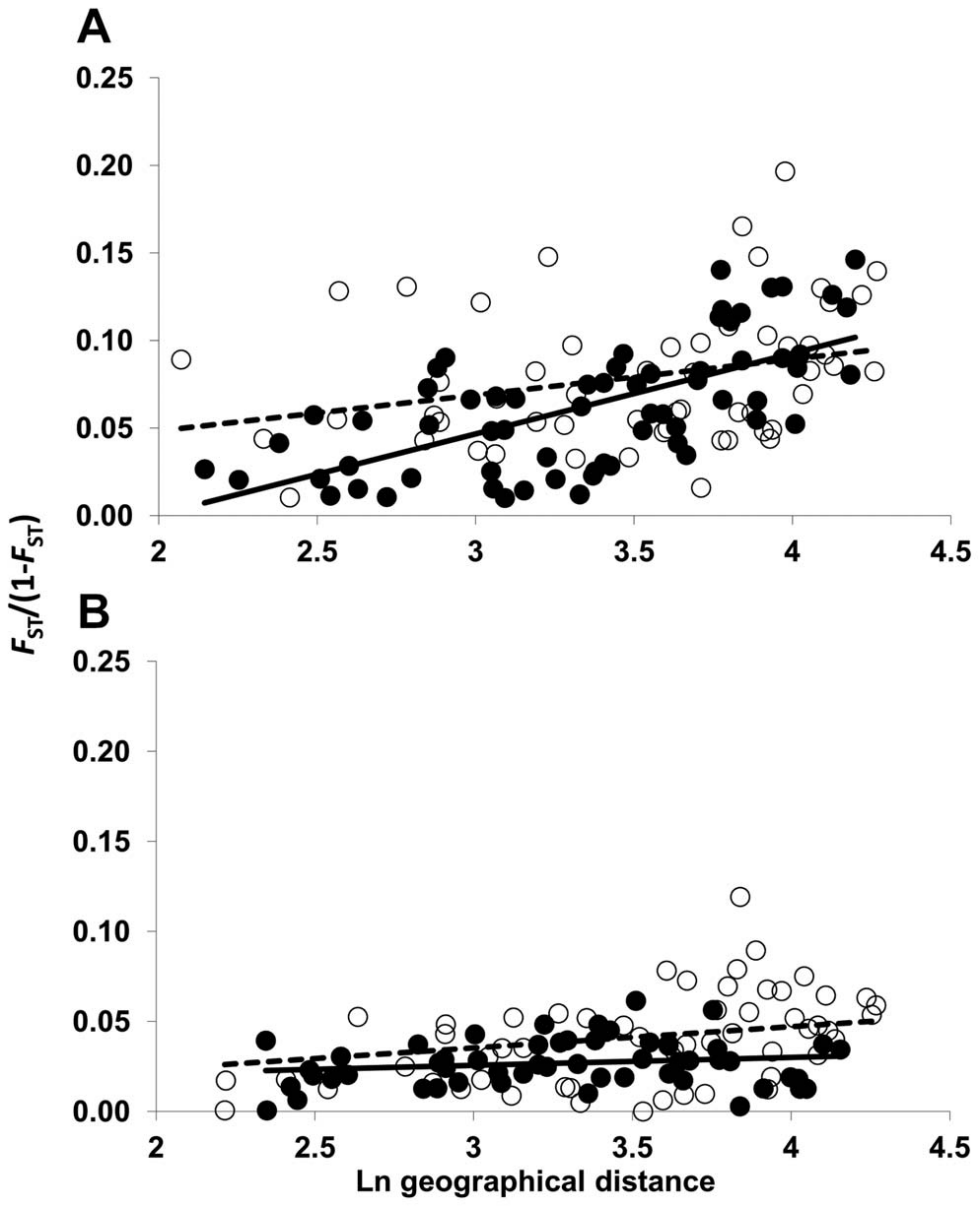
Isolation by distance (IBD). Matrix correlations between pairwise linearized *F*_ST_ values (Slatkin 1995) (y-axis) and log-transformed pairwise geographical (x-axis) distance values for *E. australis* (a) and *E. arborea*. (b). Correlation values for *E. australis* within each side of were *r* = 0.654, *p* = 0.001 and *r* = 0.282, *p* = 0.046 for Algeciras and Tangier, respectively. Correlation values for *E. arborea* within each side of were *r* = 0.17, *p* = 0.142 and *r* = 0.475, *p* = 0.004 for Algeciras and Tangier respectively. *P* values reported after 1000 random permutations Mantel tests. Black circles and white circles indicate Algeciras and Tangier pairwise comparisons, respectively. Solid lines and dashed lines represent correlation lines for Algeciras and Tangier, respectively.

Populations of *E. australis* showed significant isolation by distance (IBD) within each side of the Strait of Gibraltar (Fig. 4a). Nonetheless, a stronger correlation between genetic distance (pairwise linearized *F*_ST_) and geographical distance was obtained for Algeciras populations, as denoted by their significantly steeper correlation line (*p*-value <0.03, ANCOVA; Fig. 4a). In *E. arborea*, by contrast, a stronger correlation between genetic and geographical distances was obtained in Tangier (*p*-value <0.01, ANCOVA; Fig. 4b), whereas no significant correlation was obtained for Algeciras populations (Fig. 4b).

Regarding estimated levels of gene flow between populations (*N*_m_), both *E. australis* and *E. arborea* showed conspicuous differences between the two sides of the Strait. In *E. australis*, a significant decrease in gene flow levels was found in Tangier between pairs of populations separated less than 30 km from each other (mean *N*_m_, 95% CI: 5.08, 3.12–7.07) compared to Algeciras (9.73, 7.55–12.0), as evidenced by the non-overlapping confidence intervals. This difference vanished at distances longer than 30 km (3.86, 3.04–4.66 *vs*. 3.57, 2.96–4.11 in Tangier and Algeciras, respectively). In contrast, *E. arborea* showed similar levels of gene flow between populations at each side of the Strait, both for population pairs at distances shorter than 30 km (31.30, −1.89–68.01 *vs*. 25.47, −1.97–51.02) and longer than 30 km (7.92, 5.05–10.78 *vs*. 13.58, 6.96–20.24, in Tangier and Algeciras, respectively).

## Discussion

Habitat patchiness leads to small population size and spatial isolation, which may also cause loss of genetic diversity in those populations by genetic drift and reduced gene flow among them [3], [7]. This study illustrates the effect of an overall increase in habitat patchiness on the population genetic structure of habitat specialist species [17], [56] compared to generalists [19]. It might seem contradictory, though, that the sandstone specialist *E. australis* has higher genetic diversity values than the generalist *E. arborea* (Table 2). This is likely a consequence of their different biogeographical histories and times of origin. The two species seem to be phylogenetically close [27], [28], but *E. arborea* occupies a more derived position compared to *E. australis* in the published phylogenies [27], [28] that, despite the absence of lineage dating analysis, suggests an earlier origin for *E. australis*. This species is endemic to the western end of the Mediterranean Basin and most likely originated somewhere in this region, on either side of the Strait of Gibraltar. In contrast, *E. arborea* is a widespread, circum-Mediterranean species [30], which probably originated in eastern Africa/Arabia and reached the Strait area through recent (Pleistocene) range expansion [57]. The presumably later divergence of *E. arborea* and, particularly, a progressive loss of genetic diversity associated with its westward migration [57], [58] would account for its overall low genetic diversity values in the Strait of Gibraltar compared to those of *E. australis*, both within and among populations (Tables 1 and 2). Nonetheless, what we highlight in this study is that average levels of within-population genetic diversity (*H*_S_) and between-population gene flow levels (*N*_m_) in the sandstone specialist *E. australis* were significantly lower in Tangier (overall high sandstone patchiness) than in Algeciras (overall low patchiness), whereas no differences concerning sandstone patchiness were detected in the edaphic generalist *E. arborea* (Table 2) between both sides of the Strait.

The lower inbreeding coefficients (*F*IS) in Tangier populations of *E. australis* than in Algeciras ones (Table 2) may at first seem contradictory, since inbreeding is expected to increase with fragmentation in plant populations [3]. However, *F*_IS_ may be biased upward in large, or highly spatially structured populations because of the Wahlund effect [3], [59]. Therefore, *F*_IS_ should be considered with caution as an isolate indicator of inbreeding [7] and will not be discussed further.

Low genetic diversity values in discontinuous populations are normally coupled with high levels of genetic differentiation [60]. This study did not detect significantly higher genetic differentiation between populations (*F*_ST_) of the sandstone specialist *E. australis* in Tangier despite their significantly lower genetic diversity (*H*_S_) values in this side of the Strait (Table 2). This may be partially a consequence of the mixed origin of populations in both sides of the Strait (Fig. 3a). Nonetheless, this species showed a somewhat higher genetic structure in Tangier (three genetic clusters are predominant in Tangier *vs*. two in Algeciras, Fig. 2b). By contrast, an increase in genetic structure in Tangier was not found for *E. arborea* (Fig. 2c), probably as a consequence of its broader ecological (edaphic) niche. Populations growing outside the sandstone patches would make population connectivity in this species more similar between both Tangier and Algeciras ranges than in the sandstone specialist *E. australis*.

While significant correlations between geographical and genetic distances (i.e. significant IBD signals) were detected for the sandstone specialist *E. australis* at each side of the Strait (Fig. 4a), the IBD signal was only significant in Tangier for the generalist *E. arborea* (Fig. 4b). The wider edaphic spectrum of the latter species is one likely reason that might contribute to homogenize its genetic spatial structure under conditions of low edaphic patchiness. On the other hand, the presence of two genetic groups of populations of *E. arborea* in Tangier with a tendency of a north-south geographic structure (Fig. 2c; Fig. 3b) might account for the increase in IBD at this side of the Strait. Unlike *E. arborea*, the IBD signal somehow weakened in Tangier for *E. australis*, as reflected by the more gentle correlation slope comparisons of their correlation coefficients (Fig. 4a). This weakening of the IBD signal in Tangier populations of the sandstone specialist species is caused by strong genetic differentiation (*F*_ST_) values between pairs of geographically close populations that fall in different, isolated sandstone patches (e.g. T12, T15, T16, T19, T23; see Fig. 1), indicating low levels of gene flow between the intervening populations [61]. Restrictions on gene flow are certainly a primary consequence of spatial isolation [14], [62]. In this regard, the effect of the edaphic (sandstone) isolation in Tangier is clearly evidenced by the significant drop of relative gene flow levels (*N*_m_) between populations of the sandstone specialist *E. australis* distant less than 30 km in this region compared to Algeciras, which was not found for the edaphic generalist *E. arborea*.

High patchiness leads to genetic impoverishment and isolation of populations, which makes them more prone to (local) extinction [3], [63]. These results provide evidence of how natural habitat (sandstone) patchiness has a more marked effect on the genetic diversity and gene flow in populations of a sandstone specialist heath species (*E. australis*) than in those of an edaphic generalist (*E. arborea*). Since most endemic species in Mediterranean heathlands of the Strait of Gibraltar are sandstone specialist [21], this study allows us to infer a high vulnerability of this endemic flora to genetic erosion and local extinction of populations in the Tangier side of the Strait of Gibraltar.

## Supporting Information

Figure S1 (a, b) Log-likelihood of the seven microsatellite loci data for 23 populations of *E. australis* (a) and of the eight microsatellite loci data for 22 populations of *E. arborea* (b) given *K* clusters, obtained through 10 runs of the STRUCTURE analysis for each *K*. Corresponding Δ*K* estimation (c, d) according to Evanno *et al*. (2005) showing maximum peaks of Δ*K* values at *K = *2 and *K = *4 for *E. australis* (c), and at *K = *2 for *E. arborea* (d), indicating that those are the optimal solutions for *K* given the data.
(DOC)

Table S1 **Allele frequencies for 7 microsatellite loci in 23** ***Erica australis*** **populations.** Numbers in brackets indicate sample sizes. Number of sampled individuals (*N*) in each population is indicated in paretheses.
(DOC)

Table S2 **Allele frequencies for 8 microsatellite loci in 22** ***Erica arborea*** **populations.** Numbers in brackets indicate sample sizes. Number of sampled individuals (*N*) in each population is indicated in paretheses.
(DOC)
