# Supplementary material for: Population Genetic Structure of a Sandstone Specialist and a Generalist Heath Species at Two Levels of Sandstone Patchiness across the Strait of Gibraltar
Source: PLoS One. 2014 May 30;9(5):e98602. doi: 10.1371/journal.pone.0098602 (PMC4039479; doi:10.1371/journal.pone.0098602)
Supplement: Figure S1 — (a, b) Log-likelihood of the seven microsatellite loci data for 23 populations of E. australis (a) and of the eight microsatellite loci data for 22 populations of E. arborea (b) given K clusters, obtained through 10 runs of the STRUCTURE analysis for each K. Corresponding ΔK estimation (c, d) according to Evanno et al. (2005) showing maximum peaks of ΔK values at K = 2 and K = 4 for E. australis (c), and at K = 2 for E. arborea (d), indicating that those are the optimal solutions for K given the data. (DOC) [file pone.0098602.s001.doc]

**(a)**

**(b)**

**(c)**

**(d)**

**LnP(D)**

**Δ*K***

***K***

***K***

**Δ*K***

**LnP(D)**
