# Supplementary material for: Population Genetic Structure of a Sandstone Specialist and a Generalist Heath Species at Two Levels of Sandstone Patchiness across the Strait of Gibraltar
Source: PLoS One. 2014 May 30;9(5):e98602. doi: 10.1371/journal.pone.0098602 (PMC4039479; doi:10.1371/journal.pone.0098602)
Supplement: Table S1 — Allele frequencies for 7 microsatellite loci in 23 Erica australis populations. Numbers in brackets indicate sample sizes. Number of sampled individuals (N) in each population is indicated in paretheses. (DOC) [file pone.0098602.s002.doc]

|  | | | | | | | | | | | | | | | | | | | | | | | |
| --- | --- | --- | --- | --- | --- | --- | --- | --- | --- | --- | --- | --- | --- | --- | --- | --- | --- | --- | --- | --- | --- | --- | --- |
| **Range** | **Algeciras** | | | | | | | | | | | | **Tangier** | | | | | | | | | | |
| **Locus/**  **Population** | **A02** | **A04** | **A06** | **A08** | **A09** | **A11** | **A12** | **A13** | **A14** | **A15** | **A16** | **A17** | **T01** | **T05** | **T06** | **T10** | **T11** | **T12** | **T15** | **T16** | **T18** | **T19** | **T23** |
| **Ecoc108** | (30) | (30) | (29) | (30) | (30) | (30) | (30) | (30) | (30) | (30) | (30) | (30) | (30) | (30) | (30) | (30) | (29) | (20) | (30) | (30) | (30) | (30) | (30) |
| **135** | 0 | 0.0167 | 0 | 0 | 0 | 0 | 0 | 0 | 0.0179 | 0 | 0 | 0 | 0 | 0 | 0 | 0 | 0 | 0 | 0 | 0 | 0 | 0 | 0 |
| **145** | 0 | 0 | 0 | 0 | 0 | 0 | 0 | 0 | 0 | 0 | 0 | 0 | 0 | 0 | 0 | 0 | 0 | 0 | 0 | 0 | 0 | 0.0333 | 0 |
| **149** | 0 | 0 | 0.0172 | 0 | 0 | 0.0167 | 0 | 0 | 0 | 0 | 0 | 0 | 0 | 0 | 0 | 0 | 0 | 0 | 0 | 0 | 0 | 0 | 0 |
| **153** | 0.0167 | 0 | 0 | 0 | 0 | 0 | 0 | 0 | 0 | 0 | 0 | 0 | 0 | 0 | 0 | 0 | 0 | 0 | 0 | 0 | 0 | 0 | 0 |
| **155** | 0 | 0 | 0 | 0 | 0 | 0 | 0 | 0 | 0 | 0.0167 | 0 | 0 | 0 | 0.0333 | 0 | 0 | 0 | 0 | 0 | 0 | 0 | 0 | 0 |
| **157** | 0.0167 | 0 | 0 | 0 | 0 | 0 | 0 | 0 | 0 | 0 | 0 | 0 | 0.0333 | 0 | 0.0500 | 0 | 0 | 0 | 0.0167 | 0 | 0.0167 | 0 | 0 |
| **159** | 0.0500 | 0.0333 | 0 | 0 | 0 | 0.0333 | 0.0500 | 0 | 0.1964 | 0.0167 | 0.1167 | 0.0167 | 0.1000 | 0.0167 | 0 | 0.2500 | 0 | 0 | 0 | 0.0167 | 0 | 0.0167 | 0.0500 |
| **161** | 0.1167 | 0.1167 | 0.2759 | 0.2167 | 0.1000 | 0.1833 | 0.2667 | 0.1167 | 0.0536 | 0.0667 | 0.0167 | 0.0667 | 0.0833 | 0.1167 | 0.0333 | 0.2000 | 0.2931 | 0.0500 | 0.0667 | 0.0833 | 0.1000 | 0.1500 | 0.1333 |
| **163** | 0.2167 | 0.3500 | 0.2759 | 0.2000 | 0.0500 | 0.3500 | 0.3667 | 0.1500 | 0.4107 | 0.3500 | 0.1833 | 0.4167 | 0.1667 | 0.1833 | 0.2333 | 0.2500 | 0.2069 | 0.2000 | 0.4167 | 0.2667 | 0.1000 | 0.2500 | 0.3833 |
| **165** | 0.1667 | 0.1167 | 0.1379 | 0.1833 | 0.3667 | 0.1333 | 0.0667 | 0.1000 | 0.1429 | 0.2167 | 0.2167 | 0.1000 | 0.1167 | 0.1833 | 0.0833 | 0.0833 | 0.1379 | 0.3000 | 0.0667 | 0.1833 | 0.3167 | 0.1667 | 0.0333 |
| **167** | 0.0667 | 0.1667 | 0.0345 | 0.1500 | 0.2167 | 0.0667 | 0.1000 | 0.1500 | 0.0179 | 0.1000 | 0.1833 | 0.0667 | 0.1167 | 0.1833 | 0.0500 | 0.0333 | 0.0517 | 0 | 0.0500 | 0.0667 | 0.0500 | 0.0167 | 0.0167 |
| **169** | 0.0500 | 0.0333 | 0 | 0.0667 | 0.0333 | 0.0500 | 0.0167 | 0.0500 | 0.0179 | 0.0167 | 0.0167 | 0.0167 | 0.0167 | 0.0833 | 0.2000 | 0 | 0.0345 | 0.0500 | 0.0167 | 0 | 0.0500 | 0.0333 | 0 |
| **171** | 0.1167 | 0.0333 | 0.0517 | 0.0833 | 0.0500 | 0.1000 | 0.0333 | 0.1500 | 0.0179 | 0.0500 | 0.0833 | 0.1000 | 0.1167 | 0.0500 | 0.2000 | 0.1000 | 0.0862 | 0.2500 | 0.2667 | 0.2833 | 0.3667 | 0.1333 | 0.1833 |
| **173** | 0.0500 | 0.0333 | 0.1379 | 0.0500 | 0.0167 | 0.0167 | 0.0167 | 0.0667 | 0.0179 | 0.0167 | 0.0167 | 0.0167 | 0.0167 | 0.0167 | 0.0333 | 0.0167 | 0.0517 | 0 | 0.0167 | 0.0333 | 0 | 0.0833 | 0.1667 |
| **175** | 0.0333 | 0.0333 | 0 | 0.0500 | 0.1000 | 0.0333 | 0 | 0.0167 | 0.0536 | 0.0167 | 0.0667 | 0.1333 | 0.1333 | 0.0167 | 0.0833 | 0 | 0.0862 | 0 | 0.0167 | 0 | 0 | 0.0500 | 0.0167 |
| **177** | 0.0167 | 0 | 0 | 0 | 0.0667 | 0 | 0.0500 | 0.0500 | 0 | 0.0167 | 0.0333 | 0.0167 | 0.0667 | 0 | 0.0167 | 0 | 0.0517 | 0.0750 | 0 | 0.0167 | 0 | 0.0500 | 0 |
| **179** | 0 | 0 | 0.0172 | 0 | 0 | 0 | 0 | 0.0333 | 0.0357 | 0.0333 | 0 | 0.0167 | 0 | 0 | 0 | 0.0667 | 0 | 0 | 0 | 0.0500 | 0 | 0 | 0 |
| **181** | 0.0500 | 0 | 0 | 0 | 0 | 0 | 0 | 0.0333 | 0.0179 | 0.0500 | 0.0333 | 0 | 0.0167 | 0.0333 | 0.0167 | 0 | 0 | 0.0750 | 0.0500 | 0 | 0 | 0 | 0 |
| **183** | 0.0167 | 0 | 0.0517 | 0 | 0 | 0 | 0 | 0 | 0 | 0.0333 | 0 | 0 | 0 | 0.0833 | 0 | 0 | 0 | 0 | 0.0167 | 0 | 0 | 0 | 0 |
| **185** | 0.0167 | 0.0333 | 0 | 0 | 0 | 0 | 0 | 0 | 0 | 0 | 0 | 0.0167 | 0 | 0 | 0 | 0 | 0 | 0 | 0 | 0 | 0 | 0.0167 | 0 |
| **187** | 0 | 0 | 0 | 0 | 0 | 0.0167 | 0.0167 | 0 | 0 | 0 | 0.0333 | 0 | 0 | 0 | 0 | 0 | 0 | 0 | 0 | 0 | 0 | 0 | 0 |
| **189** | 0 | 0 | 0 | 0 | 0 | 0 | 0 | 0.0167 | 0 | 0 | 0 | 0 | 0 | 0 | 0 | 0 | 0 | 0 | 0 | 0 | 0 | 0 | 0.0167 |
| **191** | 0 | 0 | 0 | 0 | 0 | 0 | 0 | 0.0500 | 0 | 0 | 0 | 0 | 0 | 0 | 0 | 0 | 0 | 0 | 0 | 0 | 0 | 0 | 0 |
| **195** | 0 | 0.0167 | 0 | 0 | 0 | 0 | 0.0167 | 0.0167 | 0 | 0 | 0 | 0.0167 | 0.0167 | 0 | 0 | 0 | 0 | 0 | 0 | 0 | 0 | 0 | 0 |
| **199** | 0 | 0.0167 | 0 | 0 | 0 | 0 | 0 | 0 | 0 | 0 | 0 | 0 | 0 | 0 | 0 | 0 | 0 | 0 | 0 | 0 | 0 | 0 | 0 |
| **Ecoc117** |  |  |  |  |  |  |  |  |  |  |  |  |  |  |  |  |  |  |  |  |  |  |  |
| **145** | 0.0333 | 0 | 0 | 0 | 0 | 0 | 0.0167 | 0 | 0 | 0.0167 | 0 | 0.0167 | 0.0500 | 0 | 0 | 0 | 0 | 0 | 0 | 0 | 0 | 0.0167 | 0 |
| **149** | 0 | 0 | 0 | 0 | 0 | 0 | 0 | 0 | 0 | 0 | 0 | 0 | 0 | 0 | 0 | 0 | 0 | 0 | 0 | 0 | 0 | 0 | 0.0333 |
| **151** | 0.0167 | 0 | 0 | 0 | 0 | 0 | 0 | 0.0333 | 0 | 0.0167 | 0.0333 | 0 | 0 | 0.0333 | 0.1167 | 0.0167 | 0 | 0 | 0.0667 | 0.0167 | 0.0333 | 0 | 0.0333 |
| **153** | 0.3667 | 0.4333 | 0.3621 | 0.2500 | 0.3500 | 0.3000 | 0.2333 | 0.3000 | 0.2143 | 0.2500 | 0.1000 | 0.3000 | 0.2667 | 0.6500 | 0.2667 | 0.5500 | 0.3793 | 0.1750 | 0.1833 | 0.2833 | 0.3500 | 0.2000 | 0.2000 |
| **155** | 0 | 0 | 0.0172 | 0 | 0.0167 | 0 | 0.0167 | 0.0500 | 0 | 0.0167 | 0 | 0.0167 | 0 | 0.0333 | 0 | 0.0167 | 0.0172 | 0 | 0.0333 | 0.0667 | 0.1167 | 0 | 0.0333 |
| **157** | 0 | 0 | 0 | 0.0500 | 0 | 0 | 0.0167 | 0.0500 | 0 | 0 | 0.0167 | 0.0167 | 0 | 0.0167 | 0.0167 | 0 | 0.0172 | 0.0750 | 0 | 0.0167 | 0.0500 | 0 | 0.0333 |
| **159** | 0.0167 | 0 | 0 | 0 | 0.0167 | 0 | 0 | 0 | 0 | 0 | 0.0333 | 0 | 0.0500 | 0.0167 | 0 | 0 | 0 | 0 | 0.0167 | 0 | 0 | 0 | 0 |
| **161** | 0.0167 | 0 | 0.0172 | 0.0333 | 0 | 0 | 0.0500 | 0 | 0.2679 | 0.0500 | 0 | 0.0167 | 0.0500 | 0 | 0.1333 | 0.0333 | 0 | 0 | 0 | 0 | 0 | 0 | 0 |
| **163** | 0.0667 | 0 | 0 | 0.0167 | 0 | 0 | 0 | 0.0167 | 0 | 0.0333 | 0 | 0.0167 | 0.0167 | 0 | 0.0500 | 0 | 0.0172 | 0.0750 | 0 | 0 | 0 | 0 | 0.0167 |
| **165** | 0 | 0 | 0.0172 | 0.1500 | 0.1000 | 0.0667 | 0 | 0.0167 | 0 | 0.0667 | 0.0667 | 0.0167 | 0.1000 | 0 | 0.0333 | 0 | 0 | 0 | 0 | 0 | 0 | 0.0333 | 0 |
| **167** | 0.0500 | 0.0667 | 0 | 0.0500 | 0.1333 | 0.0333 | 0 | 0.0500 | 0 | 0.0167 | 0.0333 | 0 | 0.0333 | 0 | 0.0500 | 0.0167 | 0 | 0.0500 | 0.0167 | 0 | 0.0333 | 0 | 0 |
| **169** | 0.0667 | 0.0667 | 0.0517 | 0.0167 | 0.0667 | 0.0333 | 0 | 0.0333 | 0.0893 | 0.0500 | 0.0333 | 0.1167 | 0 | 0 | 0.0167 | 0 | 0.0172 | 0.0250 | 0.0167 | 0.0167 | 0 | 0.0500 | 0 |
| **171** | 0.0167 | 0.0167 | 0.0345 | 0.0167 | 0.0500 | 0.1167 | 0.0167 | 0.0333 | 0.0179 | 0.0333 | 0.0500 | 0.0167 | 0.0333 | 0 | 0 | 0.0333 | 0.0517 | 0 | 0.1167 | 0 | 0.0667 | 0.0167 | 0.0667 |
| **173** | 0.0500 | 0.0500 | 0 | 0.0667 | 0 | 0.0167 | 0.0667 | 0.0333 | 0.0714 | 0.0333 | 0.0500 | 0 | 0 | 0 | 0.0500 | 0.0333 | 0 | 0 | 0.1167 | 0.0167 | 0.0333 | 0 | 0 |
| **175** | 0 | 0 | 0 | 0.0167 | 0 | 0.0167 | 0 | 0.0167 | 0 | 0 | 0.0500 | 0.0167 | 0.0167 | 0 | 0.0167 | 0.0167 | 0.0517 | 0 | 0.0500 | 0.0500 | 0 | 0.0333 | 0.0667 |
| **177** | 0.0167 | 0.0167 | 0.0345 | 0.0167 | 0.0333 | 0 | 0.0167 | 0.0167 | 0.1071 | 0 | 0.0167 | 0.0500 | 0.0333 | 0.0167 | 0.0167 | 0.0333 | 0.0172 | 0 | 0.0167 | 0 | 0.0167 | 0.0833 | 0.1667 |
| **179** | 0.0167 | 0.0667 | 0.0517 | 0.0167 | 0 | 0 | 0.0167 | 0.0167 | 0 | 0 | 0.0333 | 0 | 0.0167 | 0.0333 | 0 | 0.0667 | 0.0517 | 0.1000 | 0.0667 | 0.1000 | 0.0333 | 0.1333 | 0.0667 |
| **181** | 0 | 0.0500 | 0.0517 | 0 | 0.0167 | 0.0333 | 0.0833 | 0.0833 | 0 | 0.0167 | 0 | 0.0333 | 0.0167 | 0.0333 | 0 | 0.0167 | 0.0517 | 0.0750 | 0 | 0.1333 | 0.0667 | 0.0333 | 0.0333 |
| **183** | 0.0500 | 0.0167 | 0 | 0 | 0 | 0 | 0.0667 | 0.0333 | 0 | 0.0333 | 0.0500 | 0.0167 | 0.0500 | 0 | 0.0167 | 0 | 0.0517 | 0 | 0.0833 | 0.0333 | 0.0167 | 0.0167 | 0.0167 |
| **185** | 0.0167 | 0 | 0.0690 | 0 | 0 | 0.0167 | 0.0500 | 0 | 0 | 0.0833 | 0 | 0.0333 | 0.0167 | 0 | 0 | 0.0167 | 0.0172 | 0.0500 | 0.0833 | 0.1167 | 0 | 0.0167 | 0.0333 |
| **187** | 0.0500 | 0 | 0.0172 | 0.0167 | 0.0167 | 0.0500 | 0 | 0 | 0.0357 | 0 | 0.0167 | 0.0333 | 0 | 0.0167 | 0 | 0.0167 | 0.0345 | 0 | 0.0500 | 0.0167 | 0.0500 | 0.1000 | 0 |
| **189** | 0.0167 | 0 | 0.1034 | 0.0333 | 0 | 0.0500 | 0.0167 | 0.0167 | 0 | 0.0833 | 0.0333 | 0.0833 | 0.0333 | 0 | 0.1000 | 0.0333 | 0.0345 | 0 | 0.0333 | 0.0167 | 0.0167 | 0.0500 | 0.0167 |
| **191** | 0.0167 | 0.0333 | 0.0172 | 0.0333 | 0 | 0 | 0.0333 | 0.0167 | 0.0357 | 0.0167 | 0.1333 | 0.0167 | 0.0167 | 0.0333 | 0 | 0 | 0.0172 | 0.0250 | 0 | 0 | 0 | 0.0667 | 0 |
| **193** | 0 | 0.0333 | 0.0517 | 0.0667 | 0.0167 | 0.0500 | 0.0500 | 0 | 0.0714 | 0 | 0.0167 | 0.0167 | 0.0333 | 0 | 0.0500 | 0 | 0.0172 | 0.0750 | 0.0167 | 0.0333 | 0 | 0.0667 | 0.0500 |
| **195** | 0.0333 | 0.0333 | 0 | 0.0500 | 0 | 0.0333 | 0.0333 | 0 | 0 | 0.0167 | 0.0500 | 0.0167 | 0.0500 | 0 | 0.0167 | 0.0167 | 0.0690 | 0.1250 | 0 | 0.0167 | 0.0167 | 0.0333 | 0 |
| **197** | 0.0167 | 0.0667 | 0 | 0.0500 | 0.0167 | 0.0167 | 0.0333 | 0 | 0 | 0 | 0.1000 | 0.0167 | 0.0500 | 0.0167 | 0.0167 | 0 | 0.0172 | 0.1250 | 0.0167 | 0 | 0 | 0.0167 | 0.0667 |
| **199** | 0 | 0 | 0.0172 | 0 | 0.0167 | 0.0167 | 0.0167 | 0.0500 | 0.0179 | 0 | 0 | 0.0333 | 0.0167 | 0 | 0 | 0 | 0 | 0 | 0 | 0.0167 | 0 | 0.0167 | 0 |
| **201** | 0.0167 | 0.0167 | 0 | 0.0167 | 0 | 0.0167 | 0.0333 | 0.0333 | 0.0179 | 0.0167 | 0 | 0.0333 | 0 | 0.0333 | 0.0167 | 0 | 0.0172 | 0 | 0 | 0.0167 | 0.0167 | 0 | 0.0333 |
| **203** | 0.0167 | 0.0167 | 0.0517 | 0.0167 | 0.0333 | 0.0167 | 0 | 0 | 0.0179 | 0.0167 | 0 | 0.0333 | 0 | 0 | 0.0167 | 0 | 0.0172 | 0 | 0 | 0 | 0.0167 | 0 | 0 |
| **205** | 0.0167 | 0.0167 | 0 | 0 | 0.0167 | 0.0333 | 0 | 0 | 0 | 0.0167 | 0.0333 | 0 | 0 | 0.0333 | 0 | 0 | 0.0345 | 0.0250 | 0.0167 | 0 | 0.0167 | 0 | 0 |
| **207** | 0 | 0 | 0 | 0 | 0 | 0 | 0 | 0.0167 | 0.0179 | 0.0167 | 0 | 0.0167 | 0.0333 | 0 | 0 | 0 | 0 | 0 | 0 | 0.0167 | 0 | 0 | 0.0167 |
| **209** | 0 | 0 | 0.0172 | 0 | 0 | 0 | 0 | 0 | 0 | 0.0333 | 0 | 0 | 0 | 0.0167 | 0 | 0.0333 | 0 | 0 | 0 | 0 | 0.0167 | 0.0167 | 0.0167 |
| **211** | 0 | 0 | 0 | 0 | 0 | 0 | 0.0167 | 0.0167 | 0.0179 | 0 | 0 | 0 | 0 | 0 | 0 | 0 | 0 | 0 | 0 | 0.0167 | 0.0167 | 0 | 0 |
| **213** | 0 | 0 | 0 | 0.0167 | 0 | 0.0167 | 0.0333 | 0.0167 | 0 | 0 | 0 | 0 | 0 | 0.0167 | 0 | 0 | 0 | 0 | 0 | 0 | 0 | 0 | 0 |
| **215** | 0 | 0 | 0.0172 | 0 | 0.0333 | 0.0167 | 0.0333 | 0 | 0 | 0 | 0 | 0 | 0.0167 | 0 | 0 | 0 | 0 | 0 | 0 | 0 | 0.0167 | 0 | 0 |
| **217** | 0 | 0 | 0 | 0 | 0.0333 | 0 | 0.0167 | 0.0167 | 0 | 0.0500 | 0 | 0.0167 | 0 | 0 | 0 | 0 | 0 | 0 | 0 | 0 | 0 | 0 | 0 |
| **219** | 0 | 0 | 0 | 0 | 0.0167 | 0.0333 | 0.0333 | 0.0333 | 0 | 0 | 0.0500 | 0 | 0 | 0 | 0 | 0 | 0 | 0 | 0 | 0 | 0 | 0 | 0 |
| **223** | 0.0167 | 0 | 0 | 0 | 0.0167 | 0 | 0 | 0 | 0 | 0.0167 | 0 | 0 | 0 | 0 | 0 | 0 | 0 | 0 | 0 | 0 | 0 | 0 | 0 |
| **229** | 0 | 0 | 0 | 0 | 0 | 0.0167 | 0 | 0 | 0 | 0 | 0 | 0 | 0 | 0 | 0 | 0 | 0 | 0 | 0 | 0 | 0 | 0 | 0 |
| **255** | 0 | 0 | 0 | 0 | 0 | 0 | 0 | 0 | 0 | 0 | 0 | 0 | 0 | 0 | 0 | 0.0333 | 0 | 0 | 0 | 0 | 0 | 0 | 0 |
| **279** | 0 | 0 | 0 | 0 | 0 | 0 | 0 | 0 | 0 | 0 | 0 | 0 | 0 | 0 | 0 | 0.0167 | 0 | 0 | 0 | 0 | 0 | 0 | 0 |
| **Ecoc132** |  |  |  |  |  |  |  |  |  |  |  |  |  |  |  |  |  |  |  |  |  |  |  |
| **191** | 0.1500 | 0.1500 | 0.2241 | 0.3500 | 0.0833 | 0.1167 | 0.0833 | 0.2833 | 0.3393 | 0.1667 | 0.1000 | 0.2500 | 0.2667 | 0.1000 | 0.4167 | 0.1833 | 0.1724 | 0.0250 | 0.1000 | 0.0333 | 0 | 0.1667 | 0.1000 |
| **193** | 0 | 0.0167 | 0 | 0.0167 | 0 | 0 | 0 | 0 | 0 | 0 | 0 | 0 | 0 | 0.0333 | 0 | 0 | 0.0172 | 0 | 0 | 0 | 0 | 0.0167 | 0 |
| **195** | 0 | 0 | 0.0172 | 0 | 0 | 0 | 0 | 0 | 0 | 0 | 0 | 0 | 0 | 0 | 0 | 0 | 0 | 0 | 0 | 0 | 0 | 0 | 0 |
| **197** | 0.0500 | 0.0167 | 0 | 0 | 0.0667 | 0.0167 | 0 | 0 | 0 | 0 | 0 | 0 | 0 | 0.0500 | 0 | 0 | 0.0172 | 0.0750 | 0 | 0.0167 | 0.1167 | 0 | 0 |
| **203** | 0 | 0 | 0 | 0 | 0 | 0 | 0 | 0 | 0 | 0.0167 | 0 | 0 | 0 | 0 | 0 | 0 | 0 | 0 | 0 | 0 | 0 | 0 | 0 |
| **205** | 0.0167 | 0 | 0 | 0.0167 | 0 | 0 | 0 | 0 | 0 | 0 | 0 | 0 | 0 | 0 | 0 | 0 | 0 | 0 | 0 | 0 | 0 | 0 | 0 |
| **207** | 0 | 0 | 0 | 0.0167 | 0 | 0 | 0 | 0 | 0 | 0 | 0 | 0 | 0 | 0.1000 | 0 | 0.0167 | 0 | 0 | 0 | 0 | 0 | 0 | 0 |
| **209** | 0 | 0 | 0 | 0.4500 | 0.5167 | 0.4833 | 0 | 0.4667 | 0 | 0 | 0 | 0 | 0.2667 | 0.4000 | 0.2833 | 0.4833 | 0 | 0 | 0.6667 | 0.0167 | 0.4000 | 0.5667 | 0 |
| **211** | 0.5000 | 0.4167 | 0.5000 | 0 | 0 | 0 | 0.4167 | 0 | 0.4107 | 0.4833 | 0.6833 | 0.6000 | 0 | 0.0833 | 0 | 0 | 0.5000 | 0.5000 | 0 | 0.5667 | 0 | 0 | 0.5333 |
| **213** | 0.0167 | 0 | 0 | 0.0167 | 0 | 0 | 0 | 0 | 0 | 0 | 0 | 0 | 0 | 0 | 0.0167 | 0 | 0 | 0 | 0 | 0 | 0.0167 | 0 | 0 |
| **215** | 0.0167 | 0.0167 | 0 | 0 | 0.0500 | 0 | 0.0167 | 0 | 0 | 0 | 0 | 0.0167 | 0 | 0 | 0.1000 | 0 | 0 | 0 | 0 | 0 | 0.0500 | 0 | 0 |
| **217** | 0.2333 | 0.3500 | 0.2586 | 0.0833 | 0.2333 | 0.3333 | 0.4833 | 0.1667 | 0.2500 | 0.3000 | 0.2000 | 0.1000 | 0.3833 | 0.1333 | 0.1167 | 0.3000 | 0.2069 | 0.4000 | 0.2000 | 0.3500 | 0.1667 | 0.2333 | 0.2500 |
| **219** | 0.0167 | 0.0167 | 0 | 0.0333 | 0 | 0 | 0 | 0.0167 | 0 | 0 | 0 | 0 | 0 | 0.0333 | 0.0167 | 0.0167 | 0 | 0 | 0.0167 | 0 | 0.0167 | 0 | 0 |
| **221** | 0 | 0 | 0 | 0.0167 | 0.0333 | 0 | 0 | 0 | 0 | 0 | 0 | 0 | 0 | 0.0500 | 0 | 0 | 0 | 0 | 0 | 0 | 0.0667 | 0 | 0.0667 |
| **223** | 0 | 0 | 0 | 0 | 0 | 0 | 0 | 0 | 0 | 0 | 0 | 0 | 0 | 0.0167 | 0 | 0 | 0 | 0 | 0 | 0.0167 | 0 | 0 | 0 |
| **225** | 0 | 0.0167 | 0 | 0 | 0 | 0 | 0 | 0.0167 | 0 | 0.0167 | 0 | 0 | 0.0167 | 0 | 0 | 0 | 0 | 0 | 0.0167 | 0 | 0 | 0 | 0 |
| **227** | 0 | 0 | 0 | 0 | 0 | 0.0167 | 0 | 0 | 0 | 0 | 0 | 0 | 0 | 0 | 0 | 0 | 0 | 0 | 0 | 0 | 0.0167 | 0 | 0 |
| **229** | 0 | 0 | 0 | 0 | 0.0167 | 0.0333 | 0 | 0 | 0 | 0 | 0 | 0 | 0.0167 | 0 | 0 | 0 | 0.0172 | 0 | 0 | 0 | 0 | 0 | 0 |
| **231** | 0 | 0 | 0 | 0 | 0 | 0 | 0 | 0 | 0 | 0.0167 | 0 | 0.0167 | 0 | 0 | 0 | 0 | 0 | 0 | 0 | 0 | 0.0167 | 0 | 0 |
| **233** | 0 | 0 | 0 | 0 | 0 | 0 | 0 | 0 | 0 | 0 | 0 | 0 | 0 | 0 | 0 | 0 | 0.0172 | 0 | 0 | 0 | 0 | 0 | 0 |
| **235** | 0 | 0 | 0 | 0 | 0 | 0 | 0 | 0 | 0 | 0 | 0 | 0 | 0.0167 | 0 | 0 | 0 | 0 | 0 | 0 | 0 | 0.0667 | 0 | 0.0333 |
| **237** | 0 | 0 | 0 | 0 | 0 | 0 | 0 | 0.0167 | 0 | 0 | 0 | 0.0167 | 0 | 0 | 0.0167 | 0 | 0.0345 | 0 | 0 | 0 | 0.0167 | 0 | 0 |
| **239** | 0 | 0 | 0 | 0 | 0 | 0 | 0 | 0.0167 | 0 | 0 | 0 | 0 | 0 | 0 | 0 | 0 | 0 | 0 | 0 | 0 | 0.0167 | 0 | 0.0167 |
| **241** | 0 | 0 | 0 | 0 | 0 | 0 | 0 | 0.0167 | 0 | 0 | 0 | 0 | 0 | 0 | 0 | 0 | 0.0172 | 0 | 0 | 0 | 0 | 0 | 0 |
| **243** | 0 | 0 | 0 | 0 | 0 | 0 | 0 | 0 | 0 | 0 | 0 | 0 | 0 | 0 | 0 | 0 | 0 | 0 | 0 | 0 | 0.0167 | 0.0167 | 0 |
| **245** | 0 | 0 | 0 | 0 | 0 | 0 | 0 | 0 | 0 | 0 | 0.0167 | 0 | 0 | 0 | 0 | 0 | 0 | 0 | 0 | 0 | 0 | 0 | 0 |
| **247** | 0 | 0 | 0 | 0 | 0 | 0 | 0 | 0 | 0 | 0 | 0 | 0 | 0.0333 | 0 | 0 | 0 | 0 | 0 | 0 | 0 | 0 | 0 | 0 |
| **249** | 0 | 0 | 0 | 0 | 0 | 0 | 0 | 0 | 0 | 0 | 0 | 0 | 0 | 0 | 0.0167 | 0 | 0 | 0 | 0 | 0 | 0 | 0 | 0 |
| **251** | 0 | 0 | 0 | 0 | 0 | 0 | 0 | 0 | 0 | 0 | 0 | 0 | 0 | 0 | 0.0167 | 0 | 0 | 0 | 0 | 0 | 0 | 0 | 0 |
| **267** | 0 | 0 | 0 | 0 | 0 | 0 | 0 | 0 | 0 | 0 | 0 | 0 | 0 | 0 | 0 | 0 | 0 | 0 | 0 | 0 | 0.0167 | 0 | 0 |
| **Ecoc137** |  |  |  |  |  |  |  |  |  |  |  |  |  |  |  |  |  |  |  |  |  |  |  |
| **207** | 0 | 0 | 0 | 0 | 0 | 0 | 0 | 0 | 0 | 0 | 0 | 0 | 0 | 0.0833 | 0.0167 | 0 | 0 | 0 | 0 | 0 | 0 | 0 | 0 |
| **215** | 0 | 0 | 0 | 0 | 0.0167 | 0.0500 | 0.0500 | 0.0333 | 0.0357 | 0 | 0.0667 | 0.0167 | 0 | 0 | 0 | 0 | 0 | 0 | 0 | 0 | 0 | 0 | 0.0167 |
| **219** | 0.0500 | 0.0667 | 0 | 0.0167 | 0.0167 | 0.0333 | 0.0333 | 0.0833 | 0.0893 | 0 | 0 | 0.0333 | 0.0500 | 0 | 0 | 0 | 0.0172 | 0 | 0 | 0 | 0 | 0 | 0 |
| **221** | 0 | 0.0667 | 0 | 0.1167 | 0.0667 | 0.0833 | 0 | 0.0333 | 0.0357 | 0 | 0.0167 | 0.0333 | 0 | 0.0167 | 0.1000 | 0.0667 | 0 | 0 | 0.0167 | 0 | 0.0167 | 0.0667 | 0 |
| **223** | 0.1667 | 0.0500 | 0.1379 | 0.2500 | 0.2167 | 0.2833 | 0.1500 | 0.2333 | 0.2143 | 0.0833 | 0.1000 | 0.1167 | 0.2000 | 0.1167 | 0.1333 | 0.4833 | 0.1207 | 0.1000 | 0.3333 | 0.3333 | 0.1500 | 0.2333 | 0.1333 |
| **225** | 0.2833 | 0.2667 | 0.1552 | 0.0833 | 0.1167 | 0.1000 | 0.4333 | 0.2000 | 0.2143 | 0.2167 | 0.1333 | 0.2833 | 0.2000 | 0.0500 | 0.2167 | 0.1667 | 0.2069 | 0.2500 | 0.2333 | 0.4500 | 0.2833 | 0.1667 | 0.2667 |
| **227** | 0.2000 | 0.1500 | 0.0345 | 0.1000 | 0.1500 | 0.2333 | 0.1167 | 0.1333 | 0 | 0.1167 | 0.1667 | 0.1167 | 0.0333 | 0 | 0.0333 | 0.0667 | 0.1034 | 0.1500 | 0.0167 | 0.0333 | 0 | 0.1000 | 0.2333 |
| **229** | 0 | 0.1000 | 0.0690 | 0.1000 | 0.0333 | 0.0833 | 0.1000 | 0.0667 | 0.0714 | 0.1000 | 0.2167 | 0.0833 | 0 | 0.2667 | 0.0333 | 0.0667 | 0.1379 | 0.1250 | 0 | 0.0167 | 0.0167 | 0.0833 | 0.0167 |
| **231** | 0.0333 | 0.0333 | 0.1379 | 0.0667 | 0.0333 | 0.0333 | 0 | 0.1000 | 0.2143 | 0.0833 | 0.0833 | 0.0500 | 0.1667 | 0.1833 | 0.1167 | 0.0500 | 0.0172 | 0.1250 | 0.0333 | 0 | 0.0667 | 0.1333 | 0 |
| **233** | 0.0833 | 0.1333 | 0.1379 | 0.0167 | 0.0500 | 0.0500 | 0.0167 | 0.0167 | 0.0714 | 0.0667 | 0.0333 | 0.0500 | 0.0500 | 0.0333 | 0.0500 | 0 | 0.0172 | 0.0250 | 0.0667 | 0 | 0.2333 | 0.0667 | 0.0167 |
| **235** | 0.0333 | 0.0167 | 0.1207 | 0.0333 | 0.0333 | 0.0167 | 0 | 0 | 0 | 0.0500 | 0.0667 | 0.0833 | 0.0167 | 0.1000 | 0.0333 | 0.0667 | 0 | 0.0250 | 0.0333 | 0.0167 | 0.0500 | 0.0333 | 0.1000 |
| **237** | 0.0500 | 0 | 0.1207 | 0.0333 | 0.2500 | 0.0167 | 0.0333 | 0.0167 | 0.0357 | 0.0333 | 0.0500 | 0.0167 | 0.0500 | 0.0833 | 0.0333 | 0 | 0.0345 | 0 | 0.0500 | 0.0167 | 0.0833 | 0.0333 | 0.0500 |
| **239** | 0.0333 | 0.0333 | 0.0345 | 0.0500 | 0.0167 | 0 | 0.0167 | 0.0167 | 0 | 0.0667 | 0.0333 | 0 | 0.1167 | 0.0167 | 0.0833 | 0 | 0.1379 | 0.0250 | 0.0833 | 0.0333 | 0.0167 | 0 | 0 |
| **241** | 0.0167 | 0 | 0.0517 | 0.0667 | 0 | 0 | 0.0333 | 0.0333 | 0 | 0.0833 | 0 | 0.0667 | 0.0667 | 0.0167 | 0.0500 | 0 | 0.0345 | 0.0750 | 0.0333 | 0 | 0.0167 | 0 | 0.0500 |
| **243** | 0.0167 | 0.0167 | 0 | 0.0167 | 0 | 0 | 0 | 0 | 0.0179 | 0.0500 | 0.0167 | 0.0500 | 0 | 0.0167 | 0.1000 | 0.0333 | 0 | 0.1000 | 0.0667 | 0 | 0.0667 | 0.0500 | 0.0333 |
| **245** | 0.0167 | 0.0500 | 0 | 0 | 0 | 0 | 0.0167 | 0.0167 | 0 | 0.0167 | 0.0167 | 0 | 0 | 0 | 0 | 0 | 0.1379 | 0 | 0.0167 | 0.0333 | 0 | 0 | 0.0167 |
| **247** | 0.0167 | 0.0167 | 0 | 0.0333 | 0 | 0.0167 | 0 | 0.0167 | 0 | 0.0167 | 0 | 0 | 0 | 0.0167 | 0 | 0 | 0.0172 | 0 | 0.0167 | 0.0333 | 0 | 0.0333 | 0.0667 |
| **249** | 0 | 0 | 0 | 0.0167 | 0 | 0 | 0 | 0 | 0 | 0.0167 | 0 | 0 | 0 | 0 | 0 | 0 | 0 | 0 | 0 | 0.0333 | 0 | 0 | 0 |
| **251** | 0 | 0 | 0 | 0 | 0 | 0 | 0 | 0 | 0 | 0 | 0 | 0 | 0.0500 | 0 | 0 | 0 | 0 | 0 | 0 | 0 | 0 | 0 | 0 |
| **253** | 0 | 0 | 0 | 0 | 0 | 0 | 0 | 0 | 0 | 0 | 0 | 0 | 0 | 0 | 0 | 0 | 0.0172 | 0 | 0 | 0 | 0 | 0 | 0 |
| **Ecoc142** |  |  |  |  |  |  |  |  |  |  |  |  |  |  |  |  |  |  |  |  |  |  |  |
| **105** | 0.4000 | 0.1500 | 0.1724 | 0.1667 | 0.0833 | 0.2000 | 0.1500 | 0.1500 | 0.2143 | 0.2000 | 0.2667 | 0.2667 | 0.0500 | 0.3167 | 0.4000 | 0.1167 | 0.0517 | 0 | 0.0667 | 0.0500 | 0.0833 | 0.0833 | 0.0500 |
| **108** | 0.3000 | 0.4333 | 0.5345 | 0.5000 | 0.5500 | 0.4167 | 0.6833 | 0.6000 | 0.3036 | 0.3667 | 0.4167 | 0.5333 | 0.7333 | 0.6167 | 0.5167 | 0.6667 | 0.8103 | 0.6500 | 0.8000 | 0.8833 | 0.7667 | 0.8333 | 0.7500 |
| **111** | 0.1167 | 0.1500 | 0 | 0.1667 | 0.1833 | 0.2500 | 0.1333 | 0.0333 | 0.0357 | 0.1833 | 0.2333 | 0.1333 | 0.0167 | 0.0333 | 0 | 0.0500 | 0 | 0.3500 | 0 | 0.0167 | 0.0333 | 0 | 0 |
| **114** | 0.1000 | 0.0833 | 0.1897 | 0.1167 | 0 | 0.0833 | 0 | 0.1000 | 0.2143 | 0.1833 | 0.0833 | 0.0333 | 0.0667 | 0 | 0 | 0.0167 | 0.0172 | 0 | 0.0333 | 0 | 0 | 0 | 0.0167 |
| **117** | 0 | 0 | 0 | 0.0500 | 0.0500 | 0 | 0 | 0.0667 | 0.2143 | 0.0667 | 0 | 0 | 0.0167 | 0 | 0.0500 | 0.1167 | 0.1034 | 0 | 0.0167 | 0 | 0 | 0 | 0.0667 |
| **120** | 0 | 0 | 0.1034 | 0 | 0 | 0 | 0 | 0 | 0 | 0 | 0 | 0 | 0 | 0 | 0.0167 | 0 | 0 | 0 | 0.0167 | 0 | 0 | 0 | 0.0500 |
| **123** | 0 | 0 | 0 | 0 | 0.1167 | 0 | 0 | 0.0333 | 0 | 0 | 0 | 0 | 0 | 0 | 0 | 0 | 0 | 0 | 0.0167 | 0 | 0 | 0 | 0.0333 |
| **126** | 0 | 0.0167 | 0 | 0 | 0.0167 | 0 | 0 | 0 | 0 | 0 | 0 | 0 | 0.0333 | 0 | 0 | 0 | 0 | 0 | 0 | 0 | 0 | 0 | 0 |
| **129** | 0 | 0 | 0 | 0 | 0 | 0 | 0 | 0 | 0 | 0 | 0 | 0 | 0 | 0 | 0 | 0 | 0 | 0 | 0 | 0 | 0.0167 | 0 | 0 |
| **132** | 0.0167 | 0.0167 | 0 | 0 | 0 | 0 | 0 | 0 | 0 | 0 | 0 | 0 | 0 | 0 | 0 | 0 | 0 | 0 | 0 | 0 | 0 | 0.0333 | 0 |
| **135** | 0 | 0.0167 | 0 | 0 | 0 | 0 | 0 | 0 | 0 | 0 | 0 | 0.0167 | 0 | 0 | 0 | 0.0167 | 0 | 0 | 0 | 0 | 0 | 0 | 0 |
| **138** | 0 | 0 | 0 | 0 | 0 | 0 | 0 | 0 | 0 | 0 | 0 | 0.0167 | 0.0167 | 0 | 0 | 0 | 0.0172 | 0 | 0 | 0 | 0 | 0 | 0.0167 |
| **141** | 0 | 0 | 0 | 0 | 0 | 0 | 0 | 0 | 0 | 0 | 0 | 0 | 0.0500 | 0.0333 | 0 | 0.0167 | 0 | 0 | 0 | 0 | 0 | 0 | 0 |
| **144** | 0 | 0 | 0 | 0 | 0 | 0.0167 | 0 | 0 | 0 | 0 | 0 | 0 | 0 | 0 | 0 | 0 | 0 | 0 | 0 | 0 | 0 | 0 | 0 |
| **147** | 0 | 0 | 0 | 0 | 0 | 0 | 0 | 0 | 0 | 0 | 0 | 0 | 0.0167 | 0 | 0 | 0 | 0 | 0 | 0 | 0 | 0 | 0.0167 | 0 |
| **150** | 0.0333 | 0 | 0 | 0 | 0 | 0 | 0 | 0.0167 | 0 | 0 | 0 | 0 | 0 | 0 | 0 | 0 | 0 | 0 | 0.0333 | 0 | 0 | 0 | 0.0167 |
| **153** | 0.0333 | 0.0167 | 0 | 0 | 0 | 0 | 0 | 0 | 0 | 0 | 0 | 0 | 0 | 0 | 0 | 0 | 0 | 0 | 0 | 0.0500 | 0 | 0.0333 | 0 |
| **156** | 0 | 0.0500 | 0 | 0 | 0 | 0 | 0 | 0 | 0 | 0 | 0 | 0 | 0 | 0 | 0 | 0 | 0 | 0 | 0.0167 | 0 | 0.0333 | 0 | 0 |
| **159** | 0 | 0.0500 | 0 | 0 | 0 | 0 | 0 | 0 | 0 | 0 | 0 | 0 | 0 | 0 | 0 | 0 | 0 | 0 | 0 | 0 | 0.0167 | 0 | 0 |
| **162** | 0 | 0 | 0 | 0 | 0 | 0.0167 | 0 | 0 | 0 | 0 | 0 | 0 | 0 | 0 | 0 | 0 | 0 | 0 | 0 | 0 | 0.0167 | 0 | 0 |
| **165** | 0 | 0 | 0 | 0 | 0 | 0 | 0.0167 | 0 | 0 | 0 | 0 | 0 | 0 | 0 | 0.0167 | 0 | 0 | 0 | 0 | 0 | 0 | 0 | 0 |
| **168** | 0 | 0 | 0 | 0 | 0 | 0 | 0.0167 | 0 | 0 | 0 | 0 | 0 | 0 | 0 | 0 | 0 | 0 | 0 | 0 | 0 | 0.0167 | 0 | 0 |
| **171** | 0 | 0.0167 | 0 | 0 | 0 | 0 | 0 | 0 | 0.0179 | 0 | 0 | 0 | 0 | 0 | 0 | 0 | 0 | 0 | 0 | 0 | 0 | 0 | 0 |
| **177** | 0 | 0 | 0 | 0 | 0 | 0 | 0 | 0 | 0 | 0 | 0 | 0 | 0 | 0 | 0 | 0 | 0 | 0 | 0 | 0 | 0.0167 | 0 | 0 |
| **189** | 0 | 0 | 0 | 0 | 0 | 0.0167 | 0 | 0 | 0 | 0 | 0 | 0 | 0 | 0 | 0 | 0 | 0 | 0 | 0 | 0 | 0 | 0 | 0 |
| **Ecoc431** |  |  |  |  |  |  |  |  |  |  |  |  |  |  |  |  |  |  |  |  |  |  |  |
| **157** | 0 | 0 | 0 | 0 | 0 | 0 | 0.0167 | 0 | 0 | 0 | 0 | 0 | 0 | 0 | 0 | 0 | 0 | 0.0500 | 0 | 0 | 0 | 0 | 0 |
| **159** | 0 | 0 | 0 | 0 | 0 | 0 | 0 | 0 | 0 | 0 | 0 | 0 | 0 | 0 | 0 | 0 | 0 | 0 | 0 | 0 | 0 | 0 | 0.0167 |
| **163** | 0.0167 | 0 | 0 | 0 | 0 | 0 | 0 | 0 | 0 | 0 | 0 | 0 | 0.0167 | 0 | 0 | 0.0333 | 0 | 0 | 0 | 0 | 0 | 0 | 0 |
| **169** | 0.4500 | 0.2667 | 0.3621 | 0.2000 | 0.1000 | 0.3167 | 0.5667 | 0.3167 | 0.2679 | 0.5167 | 0.2333 | 0.2167 | 0.0833 | 0.0167 | 0 | 0 | 0.1379 | 0.2250 | 0.0333 | 0.0500 | 0 | 0.0167 | 0.1167 |
| **173** | 0.1333 | 0.2667 | 0.0345 | 0.0833 | 0.4333 | 0.0833 | 0.1333 | 0.1333 | 0.5536 | 0.0667 | 0.2167 | 0.3667 | 0.4667 | 0.4167 | 0.2833 | 0.3500 | 0.2069 | 0 | 0.2000 | 0.0167 | 0.1667 | 0.1667 | 0.1833 |
| **179** | 0.4000 | 0.4500 | 0.6034 | 0.7000 | 0.3667 | 0.5500 | 0.2500 | 0.5000 | 0.1250 | 0.4000 | 0.5333 | 0.4167 | 0.3333 | 0.3333 | 0.5167 | 0.4500 | 0.6034 | 0.5750 | 0.6667 | 0.9333 | 0.5000 | 0.6167 | 0.6833 |
| **183** | 0 | 0 | 0 | 0.0167 | 0.0333 | 0.0167 | 0 | 0 | 0 | 0 | 0.0167 | 0 | 0.0667 | 0.2333 | 0 | 0 | 0.0345 | 0 | 0.0333 | 0 | 0.2667 | 0 | 0 |
| **185** | 0 | 0.0167 | 0 | 0 | 0.0667 | 0.0333 | 0.0333 | 0.0500 | 0.0179 | 0.0167 | 0 | 0 | 0.0333 | 0 | 0.0667 | 0.1333 | 0.0172 | 0.1500 | 0.0667 | 0 | 0 | 0.2000 | 0 |
| **187** | 0 | 0 | 0 | 0 | 0 | 0 | 0 | 0 | 0.0357 | 0 | 0 | 0 | 0 | 0 | 0 | 0 | 0 | 0 | 0 | 0 | 0 | 0 | 0 |
| **195** | 0 | 0 | 0 | 0 | 0 | 0 | 0 | 0 | 0 | 0 | 0 | 0 | 0 | 0 | 0.1333 | 0.0333 | 0 | 0 | 0 | 0 | 0.0667 | 0 | 0 |
| **Ecoc446** |  |  |  |  |  |  |  |  |  |  |  |  |  |  |  |  |  |  |  |  |  |  |  |
| **151** | 0 | 0 | 0 | 0 | 0 | 0 | 0 | 0 | 0 | 0.0167 | 0 | 0 | 0 | 0 | 0 | 0 | 0 | 0 | 0.0167 | 0 | 0.0167 | 0 | 0 |
| **153** | 0 | 0 | 0 | 0 | 0 | 0 | 0 | 0 | 0 | 0 | 0 | 0 | 0 | 0 | 0 | 0 | 0 | 0 | 0 | 0 | 0.0167 | 0 | 0.0167 |
| **155** | 0 | 0.0167 | 0 | 0 | 0 | 0 | 0.0167 | 0 | 0.1607 | 0 | 0.0333 | 0.0167 | 0.0167 | 0 | 0 | 0.0167 | 0.0690 | 0.0750 | 0.0333 | 0.0333 | 0 | 0.1000 | 0.0500 |
| **157** | 0.0833 | 0.0500 | 0.1034 | 0.0167 | 0 | 0.0500 | 0.0333 | 0 | 0.0536 | 0.1500 | 0.0167 | 0.0167 | 0 | 0 | 0 | 0.0167 | 0.0172 | 0.0500 | 0.0667 | 0.0333 | 0 | 0.0167 | 0.1000 |
| **159** | 0 | 0 | 0 | 0 | 0 | 0 | 0 | 0.0167 | 0.0357 | 0 | 0 | 0.0500 | 0 | 0 | 0 | 0 | 0.0172 | 0 | 0.0333 | 0 | 0 | 0 | 0.0167 |
| **161** | 0.0667 | 0.0333 | 0.0517 | 0 | 0 | 0.0167 | 0 | 0 | 0.0893 | 0.0500 | 0.1333 | 0.1667 | 0 | 0 | 0.0333 | 0 | 0.0345 | 0 | 0.0167 | 0 | 0 | 0 | 0.0833 |
| **163** | 0.1667 | 0.2833 | 0.1379 | 0.3833 | 0.6833 | 0.4000 | 0.4000 | 0.5667 | 0.1429 | 0.4000 | 0.4000 | 0.2667 | 0.2500 | 0.3333 | 0.2333 | 0.3667 | 0.2586 | 0.5500 | 0.2167 | 0.6000 | 0.5000 | 0.2500 | 0.3000 |
| **165** | 0.0167 | 0.0167 | 0.0862 | 0 | 0 | 0 | 0.0333 | 0 | 0 | 0.0167 | 0.0167 | 0.1000 | 0.0167 | 0 | 0 | 0 | 0.0517 | 0 | 0 | 0 | 0 | 0 | 0.0167 |
| **167** | 0.2333 | 0.2667 | 0.3276 | 0.4000 | 0.2333 | 0.3167 | 0.3000 | 0.2333 | 0.1786 | 0.0833 | 0.0833 | 0.1333 | 0.3500 | 0.4333 | 0.4167 | 0.3667 | 0.3966 | 0.2500 | 0.3167 | 0.2333 | 0.3167 | 0.3333 | 0.2667 |
| **169** | 0.1167 | 0 | 0.0345 | 0 | 0 | 0.0667 | 0.0167 | 0 | 0.1786 | 0.0667 | 0.1000 | 0.0667 | 0.0667 | 0.0167 | 0 | 0.0333 | 0.0517 | 0 | 0.0167 | 0 | 0 | 0 | 0.0500 |
| **171** | 0.1833 | 0.1333 | 0.0517 | 0.0167 | 0 | 0.0167 | 0.0500 | 0 | 0.0536 | 0.0833 | 0.0667 | 0.1167 | 0.0167 | 0.0167 | 0.0167 | 0.0167 | 0.0172 | 0 | 0.0500 | 0.0333 | 0 | 0.0167 | 0.0333 |
| **173** | 0.1333 | 0.1833 | 0.1379 | 0.1833 | 0.0833 | 0.1333 | 0.1167 | 0.1833 | 0.0536 | 0.1000 | 0.1333 | 0.0667 | 0.2833 | 0.1833 | 0.3000 | 0.1667 | 0.0517 | 0.0750 | 0.2000 | 0.0667 | 0.1500 | 0.1500 | 0.0167 |
| **175** | 0 | 0.0167 | 0.0690 | 0 | 0 | 0 | 0 | 0 | 0.0179 | 0 | 0 | 0 | 0 | 0 | 0 | 0 | 0.0172 | 0 | 0.0333 | 0 | 0 | 0.1333 | 0.0333 |
| **177** | 0 | 0 | 0 | 0 | 0 | 0 | 0.0333 | 0 | 0.0179 | 0.0167 | 0.0167 | 0 | 0 | 0.0167 | 0 | 0 | 0.0172 | 0 | 0 | 0 | 0 | 0 | 0.0167 |
| **179** | 0 | 0 | 0 | 0 | 0 | 0 | 0 | 0 | 0.0179 | 0 | 0 | 0 | 0 | 0 | 0 | 0.0167 | 0 | 0 | 0 | 0 | 0 | 0 | 0 |
| **183** | 0 | 0 | 0 | 0 | 0 | 0 | 0 | 0 | 0 | 0.0167 | 0 | 0 | 0 | 0 | 0 | 0 | 0 | 0 | 0 | 0 | 0 | 0 | 0 |
